# Computational and experimental pharmacology to decode the efficacy of *Theobroma cacao* L. against doxorubicin-induced organ toxicity in EAC-mediated solid tumor-induced mice

**DOI:** 10.3389/fphar.2023.1174867

**Published:** 2023-05-31

**Authors:** Priyanka P. Patil, Pranjal Kumar, Pukar Khanal, Vishal S. Patil, Harish R. Darasaguppe, Vishwambhar Vishnu Bhandare, Arati Bhatkande, Sudhanshu Shukla, Rajesh K. Joshi, Basanagouda M. Patil, Subarna Roy

**Affiliations:** ^1^ Indian Council of Medical Research- National Institute of Traditional Medicine, Belagavi, Karnataka, India; ^2^ Department of Pharmacology and Toxicology, KLE College of Pharmacy Belagavi, KLE Academy of Higher Education and Research (KAHER), Belagavi, Karnataka, India; ^3^ Department of Biosciences and Bioengineering, Indian Institute of Technology Dharwad, Dharwad, Karnataka, India; ^4^ PRES’s Pravara Rural College of Pharmacy Pravaranagar, Loni, Maharashtra, India

**Keywords:** cocoa, cytotoxicity, doxorubicin, organ toxicity, oxidative stress

## Abstract

**Background and objective*:*** Doxorubicin is extensively utilized chemotherapeutic drug, and it causes damage to the heart, liver, and kidneys through oxidative stress. *Theobroma cacao* L (cocoa) is reported to possess protective effects against several chemical-induced organ damages and also acts as an anticancer agent. The study aimed to determine whether the administration of cocoa bean extract reduces doxorubicin-induced organ damage in mice with Ehrlich ascites carcinoma (EAC) without compromising doxorubicin efficacy.

**Methodology*:*** Multiple *in vitro* methods such as cell proliferation, colony formation, chemo-sensitivity, and scratch assay were carried out on cancer as well as normal cell lines to document the effect of cocoa extract (COE) on cellular physiology, followed by *in vivo* mouse survival analysis, and the organ-protective effect of COE on DOX-treated animals with EAC-induced solid tumors was then investigated. *In silico* studies were conducted on cocoa compounds with lipoxygenase and xanthine oxidase to provide possible molecular explanations for the experimental observations.

**Results:** *In vitro* studies revealed potent selective cytotoxicity of COE on cancer cells compared to normal. Interestingly, COE enhanced DOX potency when used in combination. The *in vivo* results revealed reduction in EAC and DOX-induced toxicities in mice treated with COE, which also improved the mouse survival time; percentage of lifespan; antioxidant defense system; renal, hepatic, and cardiac function biomarkers; and also oxidative stress markers. COE reduced DOX-induced histopathological alterations. Through molecular docking and MD simulations, we observed chlorogenic acid and 8′8 methylenebiscatechin, present in cocoa, to have the highest binding affinity with lipoxygenase and xanthine oxidase, which lends support to their potential in ameliorating oxidative stress.

**Conclusion:** The COE reduced DOX-induced organ damage in the EAC-induced tumor model and exhibited powerful anticancer and antioxidant effects. Therefore, COE might be useful as an adjuvant nutritional supplement in cancer therapy.

## Introduction

Doxorubicin is an anthracycline antibiotic that has been indicated to deal with malignancy associated with vital organs with topoisomerase II inhibition, which cleaves the DNA of tumor cells (Thorn et al., 2011). Although doxorubicin is a potent cytotoxic agent in cancer chemotherapy, its utilization is limited due to its unwanted adverse effects, including myelosuppression, cardiomyopathy, and damage to other vital organs due to the free radicals produced by the doxorubicin metabolite (van der Zanden et al., 2021). In this regard, it is important to identify the agent to deal with the doxorubicin metabolite-activated reactive oxygen species (ROS) system without affecting the therapeutic action of doxorubicin. Cardiotoxicity caused by doxorubicin can be acute and manifest during or 2 to 3 days after administration. Acute cardiotoxicity occurs in about 11% of cases. There are substantially fewer cases of persistent doxorubicin cardiotoxicity, with an estimated incidence of 1.7%. For the majority of the time, it becomes evident after 30 days of administration of the last dose, although it can also happen 6–10 years afterward. Dosage is a major factor in doxorubicin cardiomyopathy incidence. When doxorubicin is administered at doses of 500–550 mg/m^2^, 551–600 mg/m^2^, and greater than 600 mg/m^2^, the incidence is around 4%, 18%, and 36%, respectively (Tri and Wilkins, 1978).

Previously, several reports have explained that medicinal plants possess a broad biological spectrum due to the presence of many secondary metabolites, which may act on multiple pathways involved in disease pathogenesis. In this regard, one can think to conceptualize the broad pharmacological spectral activity in managing cancer by “neutralization of the ROS system and enhancement of the pharmacological threshold of established chemotherapeutic agents,” which may be achieved by utilizing traditional medicines as they are rich in bioactive principles for multiple pharmacological activities with broad biological processes (Atanasov et al., 2021).

*Theobroma cacao* L. nibs (family Sterculiaceae), native to Central America, have been reported to contain multiple secondary metabolites (flavonoids, polyphenols, and alkaloids) and are also indicated for multiple pharmacological activities, *viz*., anti-inflammatory, anti-cancer, cardioprotective, nephroprotective, and hepatoprotective, including anti-oxidant properties (Zięba et al., 2019; Cura et al., 2021; Fanton et al., 2021; Sun et al., 2021). Due to these pharmacological properties, it can be hypothesized that nibs of *Theobroma cacao* may neutralize the doxorubicin-metabolite-mediated ROS system and have a protective effect on the heart, liver, and kidneys in the chemotherapy involving doxorubicin. In addition, it may also amplify the effect of doxorubicin as *T. cacao* itself possesses anti-cancer properties.

Hence, the present study aimed to investigate the effect of *T. cacao* nibs’ hydroalcoholic extract (COE) supplementation over doxorubicin-induced organ toxicities in solid tumor models and cell line models *in vitro* and also assess the anticancer efficacies of COE alone or in combination with doxorubicin.

## Materials and methods

### Plant collection, authentication, and extract preparation

Cocoa nibs were collected from Sirsi (14°.34′38.7984 N, 74°.58′21.288 E), India; authenticated at ICMR-NITM Belagavi, and deposited in the herbarium (voucher number RMRC-1392). The freshly collected nibs were washed under running water, chopped, and dried under shade; turned into a coarse powder; defatted with petroleum ether; macerated (1 week) in a closed container using ethanol 80% v/v; filtered, concentrated, and lyophilized; and stored in an airtight container for further use (Ruzaidi et al., 2005).

### *In vitro* assays

#### Cell culture and maintenance

All the *in vitro* studies were carried out on the A549, Ehrlich’s ascites carcinoma, and Chinese hamster ovary (CHO) cell lines. All the cell lines were procured from the National Center for Cell Sciences (NCCS), Pune. A549 cells were grown in Ham’s F12K media (AL1065-500 mL), while EAC and CHO cell lines were grown in Dulbecco’s modified Eagle’s medium (DMEM, D6429-500 mL), each supplemented with fetal bovine serum (Gibco: 10270–106; 10% v/v), penicillin (100 U/mL), and streptomycin (100 μg/mL) (penicillin–streptomycin, Sigma: P4333-100 ML). All the cells were cultured at 37°C and 5% CO_2_ in a humidified incubator.

#### Chemo-sensitivity assay

All three cell lines were used for chemo-sensitivity assay using various concentrations of COE (2.5 μg/mL to 2560 μg/mL), doxorubicin (0.125 μg/mL to 128 μg/mL), and a combination of both (1.25–1280 μg/mL COE + 0.0625–64 μg/mL doxorubicin) for 48 h, and cell viability was assayed using MTT (as explained in cell proliferation assay in the following section). The data were normalized and plotted.

#### Cell proliferation assay

All three cell lines were utilized for the cell proliferation assay (Kumar et al., 2019). Briefly, cells were treated with doxorubicin (1 μg/mL), COE (40 μg/mL), and a combination of doxorubicin (0.5 μg/mL) with COE (20 μg/mL) along with control (DMSO) for 48 h. Later, cells were detached using a trypsin–EDTA solution, collected, counted, and plated for all the assays. For the cell proliferation assay, 2000 cells per well were seeded in a 96-well plate (in triplicate). MTT (Sigma: M5655-1G) was added after 24, 48, and 96 h of seeding (for triplicate samples) for each treatment group, and after 4 h of incubation at 37°C, absorbance was recorded at 570 nm. The data were normalized and plotted.

#### Colony formation assay

The colony formation assay was performed similarly to the cell proliferation assay, except for the seeding density, i.e., 200 cells per well were seeded in a 24-well plate in duplicate and cultured for 2 weeks. Colonies with at least 50 cells were counted and plotted, and an image was captured and presented (Kumar et al., 2019).

#### Scratch assay

For the scratch assay, 0.2 × 10^6^ cells per well were seeded in a 24-well plate. Cells were treated with the aforementioned concentration of test samples, and the migration was analyzed by measuring the scratch size over various time points. The data were normalized as per the scratch size at 0 hours and plotted (Kumar et al., 2019).

### *In vivo* pharmacology

#### Ethical clearance and animal procurement

The experiment was performed after obtaining ethical clearance from the IAEC at ICMR-NITM, Belagavi for the use of laboratory animals (approval number: IAEC/ICMR-NITM BGM/2019/3). Healthy female Balb/c mice (22–25 g) were procured from a registered CPCSEA supplier and maintained at 23°C ± 2°C temperature, 50% ± 5% humidity, and a 12/12 h light-dark cycle. Animals had ad libitum access to food and water throughout the experiment.

#### Tumor cell transplantation and grouping of animals

The Ehrlich ascites carcinoma cells-carrying donor mice were obtained from *Invivo* biosciences, Bangalore, India, and were maintained and propagated by serial intraperitoneal transplantation (1×10^6^ cells) into healthy mice in an aseptic environment and propagated for 14 d. Ehrlich ascites carcinoma cells were received from donor mice and suspended in sterile saline. A viable number of cells (∼2 × 10^6^ cells) were injected subcutaneously into the thigh region of the right hind limb (Gothoskar and Ranadive, 1971; Elsherbiny et al., 2016).

In the present study, a total of 80 animals were randomly divided into five groups (n = 16), of which group 1 served as normal (n = 16) and the rest were induced tumors by injecting EAC cells (2×10^6^) (suspended in 0.2 mL saline/mice) subcutaneously. The tumor size of all EAC-inoculated mice crossing the 50 mm^3^ limit was considered day 0 (11th day after implantation), and treatment was initiated. Tumor size was measured using a Vernier caliper every week and calculated using the following equation as mentioned by Schirner et al. (1998).

Tumor volume=Longest diameter X Shortest width X 0.5

The study included the animals grouping as *Normal*: vehicle (2% gum acacia OD); *EAC*: vehicle (2% gum acacia *p. o.*, OD); *DOX*: EAC + doxorubicin 4.91 mg/kg, *i. p.*, *q. w.*; *COE200*: EAC + Cocoa 200 mg/kg, *p. o.*, OD; and *COE200+ DOX*: EAC + doxorubicin 4.91 mg/kg, *i. p.*, *q. w.* + cocoa 200 mg/kg, *p. o.*, OD.

The treatment was carried out for 21 days, and six animals from each group were randomly separated. Blood was collected by the retro-orbital route separately for hematological and biochemical analysis. Furthermore, animals were euthanized with an overdose of ketamine to collect the tumor mass and vital organs (heart, liver, and kidneys) for antioxidant and histopathological studies. However, the rest of the animals (n = 10 per group) were kept for survival analysis for up to 60 days. The mean survival time was calculated as explained by Elsherbiny et al. (2016) as follows:

$$MST=\frac{\Sigma \;Survival\;time\;\left(days\right)\;ofeach\;mouse\in agroup}{Total\;numberofmice}.$$

$$Increased\;life\;span\left(\%\right)=\frac{MST\;of\;treated\;mice}{MST\;of\;the\;cancer\;control\;group}X100.$$

#### Measurements and methods

As mentioned previously, body weight and tumor size were measured once a week. A total of six animals from each group were selected randomly, and blood, vital organs, and tumor mass were collected as previously mentioned. Furthermore, we evaluated multiple biochemical parameters, i.e., creatinine kinase-MB, lactate dehydrogenase, aspartate aminotransferase (AST), alanine aminotransferase (ALT), alkaline phosphatase (ALP), creatinine, and urea using commercially available kits (Biosystems) using a semi-automated analyzer (A15 Biosystems). In addition, we evaluated the anti-oxidant biomarkers, i.e., catalase (Claiborn, A., 1985), SOD (Misra and Fridovich, 1972), GSH (Sedlak and Lindsay, 1968), and LPO (Ohkawa et al., 1979). The hematological parameters such as complete blood counts (RBC, WBC, and platelets), mean corpuscular volume (MCV), mean corpuscular hemoglobin concentration, % Hb mean corpuscular hemoglobin, mean cell hemoglobin concentration, and packed cell volume were measured using an automatic hematology analyzer (Erba H560).

For histopathological analysis, the tissue was sectioned, and the samples (heart, liver, kidney, and tumor) were fixed in formalin (10%) and stained using hematoxylin and eosin. The samples were observed under a light microscope (Olympus BH-2, Olympus, Tokyo, Japan).

#### Molecular modeling studies

The anticancer agent doxorubicin induces hepatic and renal toxicity, mainly due to oxidative stress. Our experimental study showed that cacao extracts have significant potential in reducing the oxidative stress triggered by doxorubicin. Therefore, to gain detailed structural insights into this mechanism, we proposed screening phytocompounds from *T. cacao* against two important enzymes: lipoxygenase (LOX) and xanthine oxidase (XO), which are the key players in producing oxidative stress (Chung et al., 1997; Czapski et al., 2012; Kostić et al., 2015).

In this study, phytocompounds from *T. cacao* were selected, and their structures (in SDF format) were retrieved from the PubChem small-molecular databases. The virtual screening was performed using an automated POAP pipeline based on the AutoDock Vina interface (Samdani and Vetrivel, 2018; Patil et al., 2022). The structures of all the ligands were converted to 3D format and subjected to energy minimization for 5000 steps using the default LigPrep parameters in the POAP pipeline. The structures of receptors (LOX: 1N8Q and XO: 3AM9) have been prepared by removing water molecules and other heteroatoms that were used to obtain the crystal structures. The missing loop regions in the structures were built using the modeler interface in Chimera 1.15. We used the CastP tool to predict the largest and best possible binding pocket in both receptors, namely, LOX and XO. The parameters used for the virtual screening of phytochemicals against LOX and XO were selected from earlier similar studies (Patil et al., 2022b). The docked conformations obtained were ranked by the binding energy values, the intermolecular interactions were analyzed using Chimera, and publication-quality figures were generated in PyMOL. The best identified docked complex of phytocompounds for each of the receptors, i.e., LOX and XO, was further subjected to all-atom MD simulation in an explicit solvent. The chlorogenic acid (CHL) and 8,8-methylenebiscatechin (MBC) formed much more stable complexes with the least binding energy (−6.4 kcal/mol and −11.3 kcal/mol, respectively) and expressed stable non-bonded interactions at the conserved binding site region. Therefore, we have considered these two complexes, namely, LOX-CHL and XO-MBC, for further molecular dynamics simulations. To investigate the structural stability of LOX-CHL and XO-MBC complexes, we used all atom-explicit MD simulation methods using the GROMACS 2021.5 software package. The partial charges of both ligands MBC and CHL were generated using the antechamber module of AmberTools18. The Amber ff99SBildn force field was used to build the topology files. The complexes were solvated in a cuboidal box (10Å) with the TIP3P water model using periodic boundary conditions, and charges over the systems were neutralized by adding the required number of counterions such as Na+/Cl-. The topology and coordinate files for both these complexes were generated and converted to a Gromacs-compatible file format using ParmEd. The prepared systems were subjected to energy minimization using the steepest descent method, followed by a conjugate gradient. Systems were equilibrated using canonical NVT and isothermic–isobaric NPT ensembles for a period of 1 ns. The unrestrained production MD run was further continued for 100 ns, and trajectories were recorded every 2 fs. The parameters used to perform MD simulations were adopted from earlier similar studies (Khanal et al., 2022). The trajectories obtained were analyzed for structural stability and their intermolecular interactions, followed by reimaging the PBC conditions. The inbuilt Gromacs tools like gmxrms, gms rmsf, and gmx gyrate were used to analyze structural stability, and other required tools were used for specific analysis wherever required, such as CPPTRAJ and LigPlus. The plots were generated using Grace 5.1.25.

#### Statistical analysis

Data were expressed as mean ± SEM. The mean difference between groups was analyzed using one-way ANOVA followed by Tukey’s post-hoc test with GraphPad Prism *ver*. 5. The difference in mean of the group was considered to be significant if *p* < 0.05.

## Results

### *In vitro* assays

**FIGURE 1:**
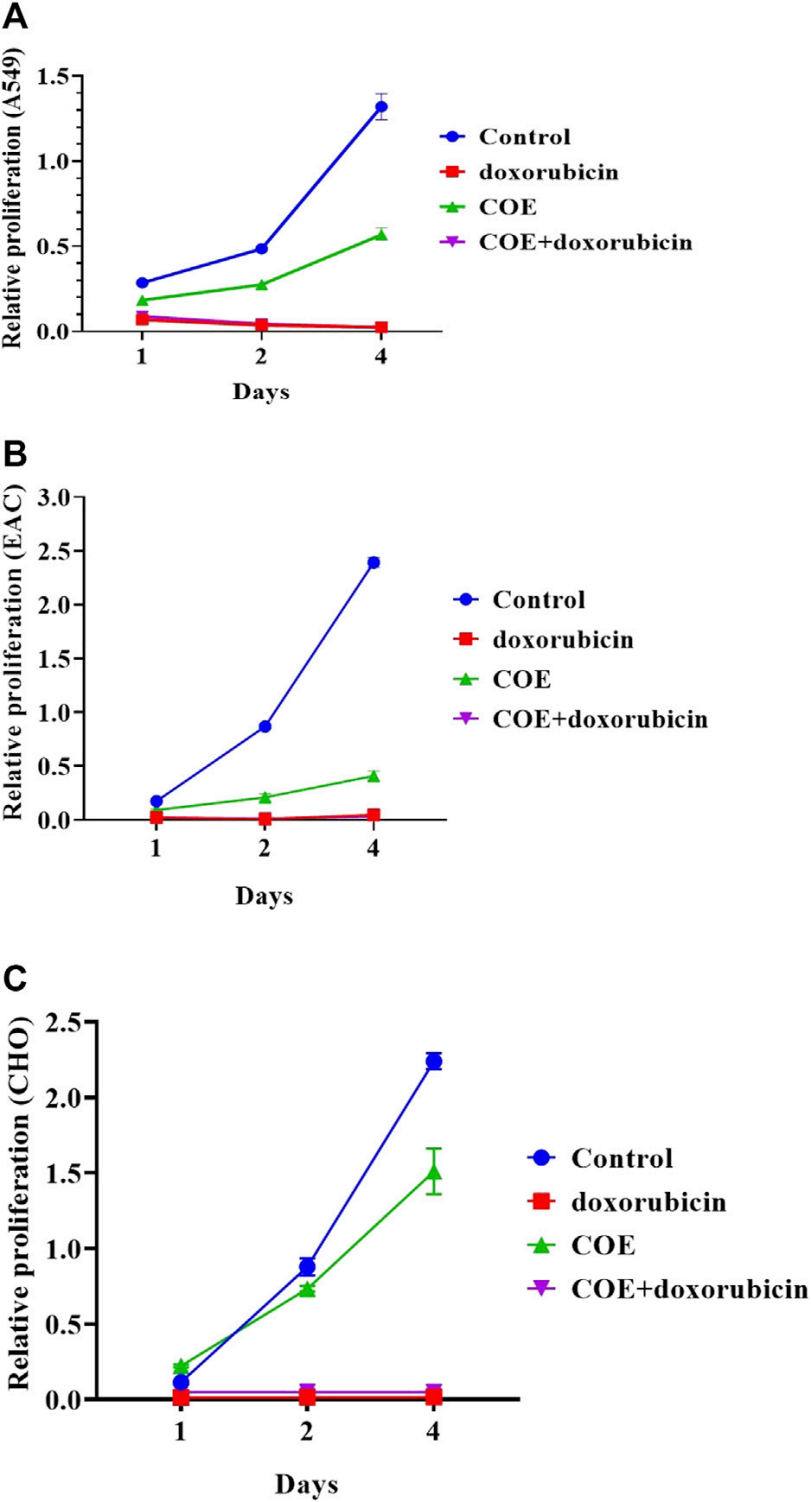
Effect of doxorubicin and COE treatment on relative cell proliferation in **(A)** A549, **(B)** EAC, and **(C)** CHO cell lines.

**FIGURE 2:**
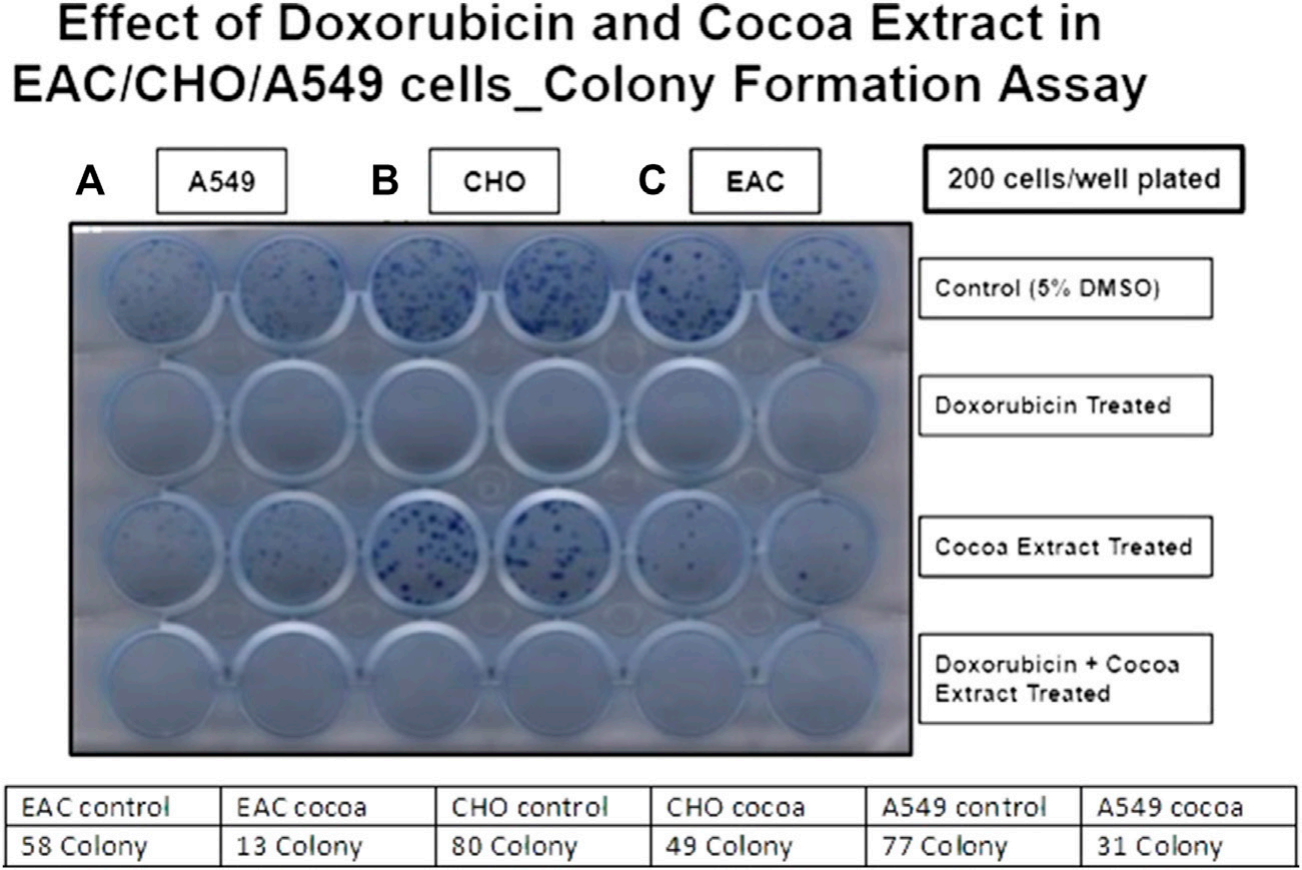
Effect of doxorubicin and COE treatment on colony formation in **(A)** A549, **(B)** CHO, and **(C)** EAC cell lines.

We examined the effect of COE on cellular physiology, such as cell proliferation and colony formation assays. In a cell proliferation assay, COE-treated cells showed reduced viability compared to the control in all three cell lines (A549, EAC, and CHO). However, the treatment with the chemotherapeutic drug, doxorubicin, was more effective than the COE-independent treatment. As expected, COE co-treatment with doxorubicin was more potent than COE-independent treatment. However, the effectiveness of COE and doxorubicin co-treatment was in a similar pattern compared to that of doxorubicin-independent treatment (Figure 1). Similarly, the colony formation assay resulted in reduced colony formation capability on COE treatment compared to control (the number of colonies was much higher in control vs. COE treatment for A549 (77 vs. 31), EAC (58 vs. 13), and CHO (80 vs. 49) cells). However, the treatment of doxorubicin alone or in combination with COE resulted in complete inhibition of colony formation capability, which is due to the lethal effect of doxorubicin (Figure 2).

**FIGURE 3:**
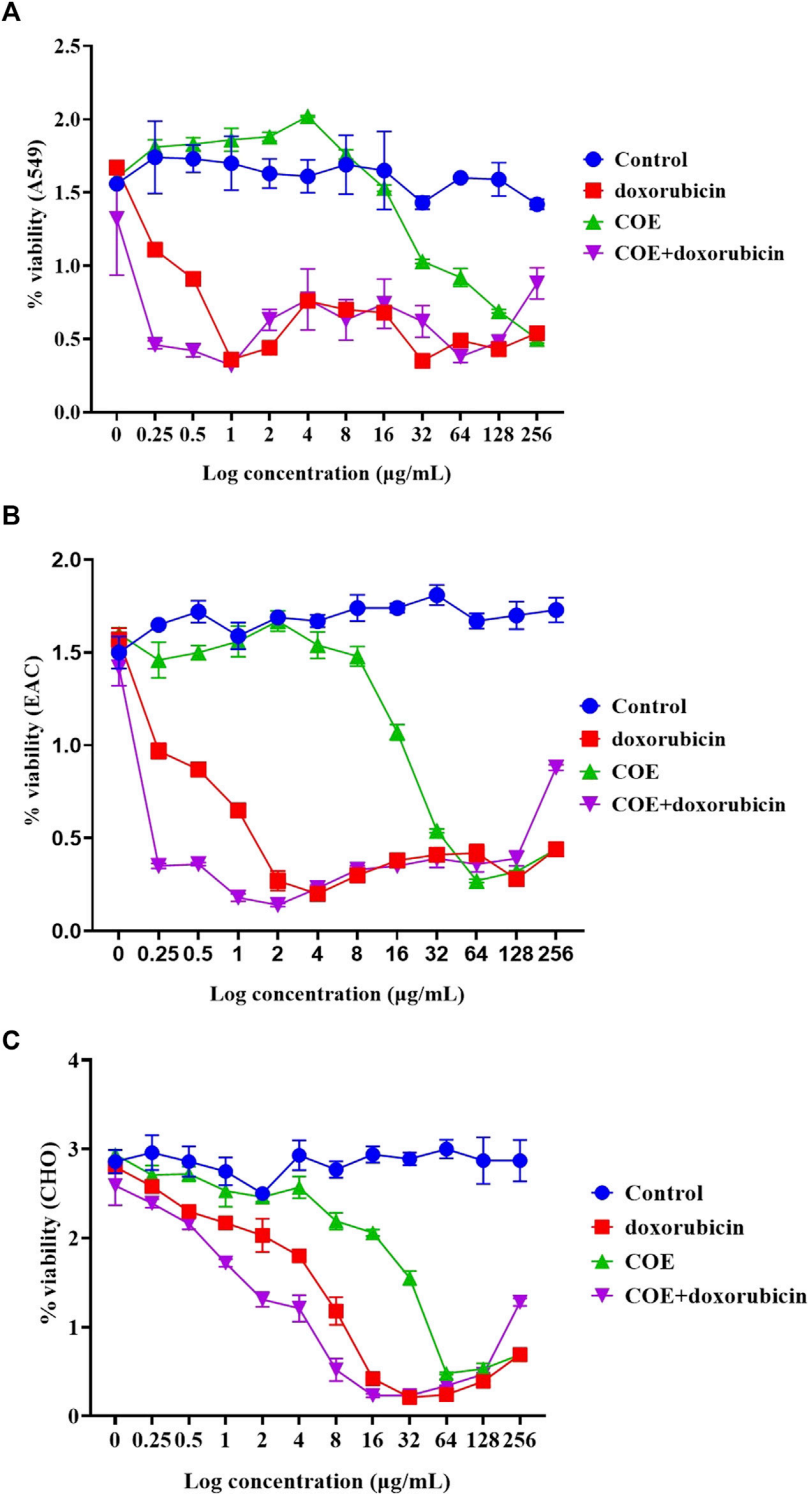
Effect of doxorubicin and COE treatment on chemo-sensitivity assays in **(A)** A549, **(B)** EAC, and **(C)** CHO cell lines.

Similarly, the chemo-sensitivity assay showed that there was a similar run in percentage viability in the control group. In COE and doxorubicin-independent treatments, there was a decrease in the percentage viability in A549, EAC, and CHO cell lines. However, it was observed that doxorubicin alone was more potent than COE-independent treatment. In addition, within the lower concentration of doxorubicin and COE treatments, the percentage viability was reduced in all the cell lines compared to the rest of the treatments, i.e., A549 (log concentration ∼0.5 μg/mL), EAC (log concentration ∼2 μg/mL), and CHO (log concentration ∼8 μg/mL) (Figure 3).

**FIGURE 4:**
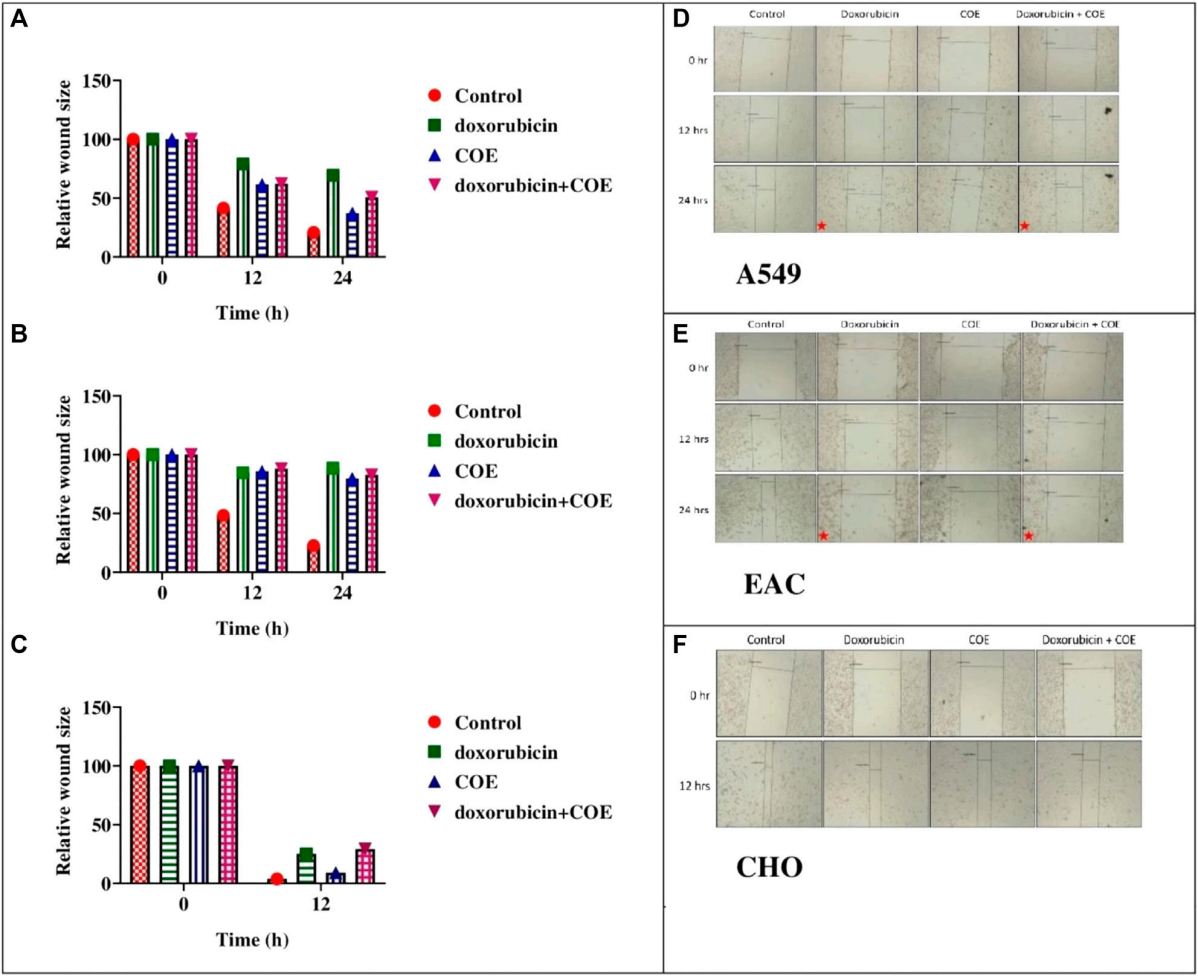
Effect of doxorubicin and COE treatment on relative scratch in **(A)** A549, **(B)** EAC, and **(C)** CHO cell lines and effect of doxorubicin and COE treatment on relative scratch in **(D)** A549, **(E)** EAC, and **(F)** CHO cell lines. Represents cell under cell death.

The scratch assay was performed to evaluate the effect of COE on migration. In A549, EAC, and CHO cell lines, the generated scratch size (at 0 h) started decreasing gradually over time in the control group. However, doxorubicin treatment led to cell death and resulted in a relatively higher scratch size at 12 and 24 h compared to the control. Interestingly, the COE treatment showed higher scratch size, i.e., a reduction in migration with time compared to the control. COE co-treatment with a lower doxorubicin concentration showed a greater reduction in migration (Figure 4A). The CHO cell line scratch closure was much faster than that of the other two cell lines, which allowed us to collect data for up to 12 h only (Figure 4B).

### *In vivo* pharmacology

#### Effect of COE treatment on survival

**FIGURE 5:**
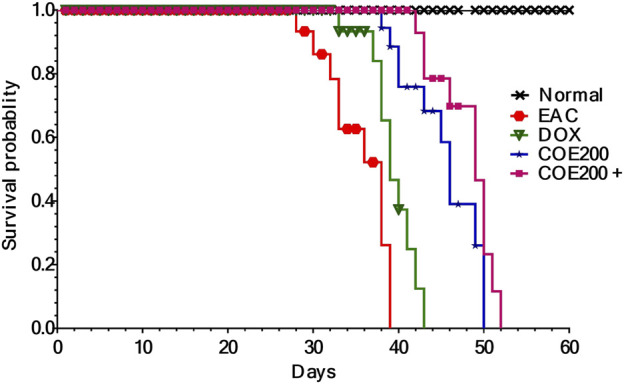
Kaplan–Meier survival curve for COE- and doxorubicin-treated groups. There was a significant difference (*p* < 0.001) in Mantel–Cox log-rank between the groups.

There was no death observed in the normal group throughout the study. However, deaths were observed from the 28th to the 39th day in the EAC group. Furthermore, treatment with COE and doxorubicin has shown an increased lifespan, and it was maximal within the COE200+ DOX group (Figure 5). The log-rank (Mantel–Cox) test reflected the increased lifespan of COE-treated animals in combination with doxorubicin compared to the rest of the animals with χ2 = 185.8 and Df = 4.

#### Effect of COE on body weight, tumor weight, and tumor size

**FIGURE 6:**
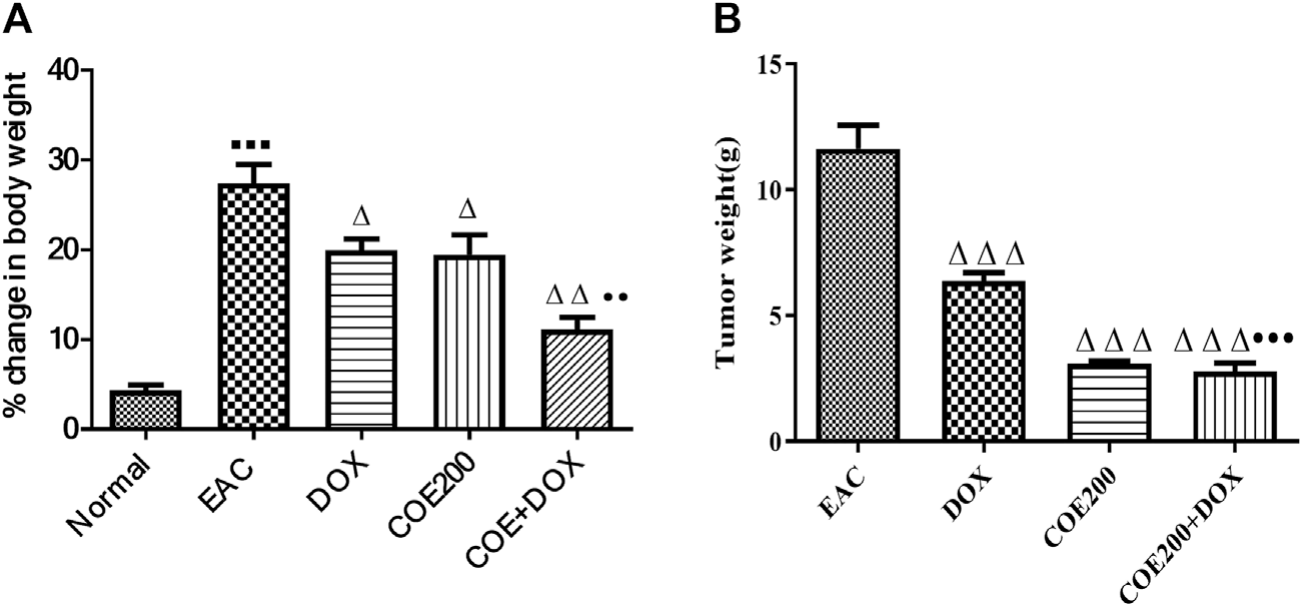
Effect of COE on **(A)** percentage change in body weight and **(B)** tumor weight (g) in solid Ehrlich tumor-bearing mice after 21 days of treatment. All values are expressed as the mean ± SEM (n = 6). One-way analysis of variance (ANOVA) followed by Tukey’s test; *p* < 0.001, compared with normal; ^Δ^ *p* < 0.05 and ^Δ^ ^Δ^ ^Δ^ *p* < 0.001, compared with EAC; ••*p* < 0.01 compared with DOX.

**FIGURE 7:**
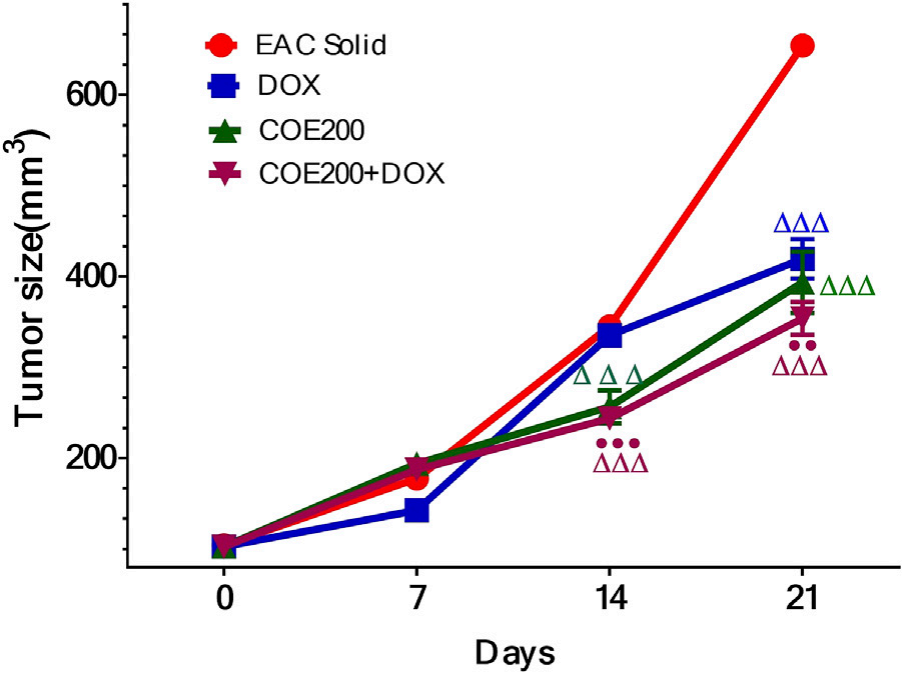
Tumor volume (mm^3^) in solid Ehrlich tumor-bearing mice. All values are expressed as a mean ± SEM (n = 6). One-way analysis of variance (ANOVA) followed by Tukey’s test; *p* < 0.001, compared with normal; ^Δ^ *p* < 0.05 and ^Δ^ ^ΔΔ^ *p* < 0.001, compared with EAC; ^●●^ *p* < 0.01 compared with DOX.

There was a significant increase in percentage change in body weight (*p* < 0.001) within EAC compared to normal, which was significantly reversed in all treatment groups (Figure 6). In addition, the combined action of COE and doxorubicin significantly decreased the change in body weight compared to doxorubicin alone (Figure 6). In addition, there was a significant decrease (*p* < 0.001) in tumor weight with doxorubicin and COE-independent and combination treatment. Furthermore, the combined action of COE and doxorubicin significantly decreased tumor weight compared to the DOX group. Likewise, there was a significant decrease (*p* < 0.001) in the progression of tumor size observed from the 14th day of treatment in COE treatment groups. Furthermore, on the 21st day, there was a significant decrease (*p* < 0.001) in tumor size in all treatment groups compared to EAC, although the combination group showed a significant reduction (*p* < 0.01) in tumor size compared to the independent DOX group (Figure 7).

#### Effect on hematological parameters

There was a significant decrease in hemoglobin (*p* < 0.001) in EAC (9.2% ± 0.15%) compared to normal (13.83% ± 0.68%), which was significantly increased (*p* < 0.01) with COE-independent treatment. A further decrease in hemoglobin in the DOX group (7.9% ± 0.18%) was observed, which was significantly increased in the combination group (10.77% ± 0.20%) compared to DOX. Furthermore, there was a significant increase (*p* < 0.001) in WBC count (51.02 ± 1.5 cell/cm) in EAC compared to normal (27.21 ± 2.1 cell/cm), which was significantly decreased (*p* < 0.001) in all treatment groups compared to EAC (Table 1). In addition, there was a significant decrease in RBC and platelet count in EAC (*p* < 0.001). These cell counts were further decreased after doxorubicin treatment. The reduction in the cell count was reversed in the COE treatment groups. There was a significant decrease (*p* < 0.001) in PCV, MCHC, and lymphocytes compared to normal, which were observed to be significantly improved (*p* < 0.005–0.001) with doxorubicin and COE treatment, independent and in combination. In contrast, there was a significant increase (*p* < 0.001) in neutrophil, eosinophils, monocytes, and MCH (*p* < 0.01) within EAC compared with normal, which was ameliorated with independent or combination of doxorubicin and COE (Table 1).

**TABLE 1:** Effect of doxorubicin and COE treatment on hematological parameters.

Groups	Normal	EAC	DOX	COE 200	COE 200+ DOX
**Hb (% g)**	13.83 ± 0.68	9.20 ± 0.15^▀^^▀^^▀^	7.90 ± 0.18	11.33 ± 0.13^▲▲^	10.77 ± 0.20^●●●^
**WBCs (cell/cmm)**	27.21 ± 2.10	51.02 ± 1.50^▀^^▀^^▀^	25.56 ± 1.70^▲▲▲^	33.91 ± 0.10^▲▲▲^	29.77 ± 1.40^▲▲▲^
**RBC million (cell/cmm)**	8.79 ± 0.20	5.00 ± 0.20^▀^^▀^^▀^	4.23 ± 0.20	7.40 ± 0.00^▲▲▲^	6.03 ± 0.20^▲●●●^
**Platelet count (cell/cmm)**	1030.00 ± 20.90	789.00 ± 20.00^▀^^▀^^▀^	645.60 ± 16.20^▲▲^	1007.00 ± 23.80^▲▲▲^	870.40 ± 35.60^●●●^
**PCV (%)**	54.78 ± 1.40	27.37 ± 2.20^▀^^▀^^▀^	32.23 ± 1.00	36.97 ± 0.70^▲▲▲^	39.63 ± 0.40^▲▲▲●●^
**MCV (fL)**	51.52 ± 0.80	52.40 ± 0.20	51.43 ± 0.30	50.37 ± 0.06^▲^	51.37 ± 0.20
**MCHC (gm/dL)**	31.75 ± 0.70	24.12 ± 0.70^▀^^▀^^▀^	31.18 ± 0.3^▲▲▲^	32.03 ± 0.40^▲▲▲^	32.05 ± 0.50^▲▲▲^
**Lymphocytes (%)**	74.50 ± 0.60	11.67 ± 1.05^▀^^▀^^▀^	19.67 ± 0.60^▲▲▲^	32.00 ± 1.00^▲▲▲^	44.50 ± 1.50^▲▲▲●●●^
**Neutrophils (%)**	21.83 ± 0.80	80.83 ± 1.60^▀^^▀^^▀^	63.00 ± 0.40^▲▲▲^	59.83 ± 0.90^▲▲▲^	54.17 ± 1.70^▲▲▲●●●^
**Eosinophils (%)**	0.66 ± 0.20	2.50 ± 0.20^▀^^▀^^▀^	1.50 ± 0.20^▲^	1.80 ± 0.10	1.30 ± 0.20^▲▲^
**Monocytes (%)**	0.30 ± 0.20	5.16 ± 0.70^▀^^▀^^▀^	2.50 ± 0.40^▲▲^	3.16 ± 0.10^▲^	2.16 ± 0.40^▲▲^
**MCH(pg)**	16.45 ± 0.20	17.90 ± 0.10^▀^^▀^	17.10 ± 0.20	15.52 ± 0.30^▲▲▲^	15.77 ± 0.30^▲▲▲●^

All values are expressed as a mean ± SEM (n = 6). One-way analysis of variance (ANOVA) followed by Tukey’s test. ^▀^^▀^p < 0.01.

^▀^^▀^^▀^p < 0.001, compared with normal.

^▲^ *p* < 0.05.

^*▲▲*^ *p* < 0.01.

^*▲▲▲*^ *p* < 0.001, compared with EAC.

^*●●*^ *p* < 0.01.

^*●●●*^ *p* < 0.001 compared with DOX.

#### Effect of COE on cardiac, hepatic, and kidney biomarkers

In the present study, we observed a significant increase (*p* < 0.001) in CK-MB, LDH, ALT, AST, ALP, creatinine, and BUN levels within the EAC groups. Furthermore, these biomarkers were significantly increased (*p* < 0.05–0.001) in the DOX group. In contrast, these markers were significantly (*p* < 0.05–0.001) ameliorated in the COE group. Likewise, in the combination treatment group, significant decreases in these parameters were observed when compared to the DOX group (Table 2).

**TABLE 2:** Effect of COE treatment on cardiac, Hepatic, and kidney biomarkers.

Groups	Normal	EAC	DOX	COE200	COE200+ DOX
**CK-MB(U/L)**	170.2 ± 1.6	251.5 ± 2.4 ^▀^^▀^^▀^	360 ± 2.6^▲▲▲^	236.8 ± 1.5^▲▲^	255.5 ± 3.2^●●●^
**LDH(U/L)**	1955 ± 14.6	4268 ± 22.5^▀^^▀^^▀^	5264 ± 20.8^▲▲▲^	2654 ± 15.5^▲▲▲^	4176 ± 18.3^▲●●●^
**AST(U/L)**	217.7 ± 16.3	620.3 ± 21.2^▀^^▀^^▀^	898.7 ± 25.4^▲▲▲^	512.2 ± 17.2^▲▲^	547.7 ± 7.9 ^▲▲^^●●●^
**ALT(U/L)**	75.5 ± 2.2	150.7 ± 2.9^▀^^▀^^▀^	234.8 ± 5.8^▲▲▲^	80 ± 6.7^▲▲▲^	124.5 ± 1.4^▲▲●●●^
**ALP(U/L)**	20.83 ± 0.4	37.83 ± 2.6^▀^^▀^^▀^	46.67 ± 1.0^▲▲^	29.33 ± 1.1^▲▲^	39.5 ± 2.0^●^
**Creatinine (mgs %)**	0.125 ± 0.0	0.28 ± 0.0^▀^^▀^^▀^	0.32 ± 0.0^▲^	0.23 ± 0.0^▲▲^	0.2383 ± 0.0^▲●●●^
**BUN (mgs %)**	30.83 ± 0.9	77.33 ± 0.9^▀^^▀^^▀^	87.67 ± 1.6^▲▲▲^	55.67 ± 0.9^▲▲▲^	70.17 ± 1.64^▲▲●●●^

All values are expressed as a mean ± SEM (n = 6). One-way analysis of variance (ANOVA) followed by Tukey’s test ^▀^^▀^^▀^p < 0.001, compared with normal.

^*▲*^ *p* < 0.05.

^*▲▲*^ *p* < 0.01.

^*▲▲▲*^ *p* < 0.001, compared with EAC.

^*●*^ *p* < 0.05.

^*●●*^ *p* < 0.01.

^*●●●*^ *p* < 0.001, compared with DOX.

#### Effect of COE on antioxidant biomarkers

There was a significant increase in the LPO level (*p* < 0.001) in the heart, liver, and kidney within EAC animals compared to normal, which was significantly reversed with COE treatment. Furthermore, there was a significant increase in LPO (*p* < 0.001) in the DOX group compared to the EAC group, which was reversed with combination treatment. Similarly, there was a significant decrease (*p* < 0.01, 0.001) in cardiac, hepatic, and kidney GSH, SOD, and CAT activities compared to normal. Doxorubicin-independent treatment had no significant influence over GSH, SOD, and CAT activities in the heart, liver, and kidney. However, COE treatment groups significantly ameliorated (*p* < 0.05–0.001) their GSH, SOD, and CAT activity in the heart, liver, and kidney compared to the EAC group. In addition, the combined action of COE with doxorubicin significantly (*p* < 0.05–0.001) influenced GSH level, SOD, and CAT activity in the heart, liver, and kidney (Table 3).

**TABLE 3:** Effect of administration of COE on doxorubicin-induced depletion of LPO, GSH, SOD, and CAT levels in heart, liver, and kidney tissues of mice.

Tissue	Treatment	LPO (nano-moles/mg of protein)	GSH (µMol/mg protein)	SOD (units/mg of protein)	CAT(units/mg of protein)
**Heart**	**Normal**	50.77 ± 1.7	26.29 ± 3.1	196.1 ± 2.7	0.57 ± 0.05
	**EAC**	177.3 ± 4.1 ^▀^^▀^^▀^	17.51 ± 1.1 ^▀^^▀^	76.87 ± 3.7 ^▀^^▀^^▀^	0.39 ± 0.02^▀^^▀^
	**DOX**	229 ± 3.08^▲▲▲^	14.99 ± 0.7	77.07 ± 5.9	0.31 ± 0.02
	**COE200**	148.3 ± 3.3^▲▲▲^	25.56 ± 1.05^▲^	111.5 ± 7.2 ^▲▲^	0.55 ± 0.01^▲^
	**COE200+ DOX**	163.6 ± 1.9^▲●●●^	22.75 ± 0.8^●^	105.4 ± 9.0^▲●^	0.49 ± 0.02^●●^
**Liver**	**Normal**	86.5 1 ± 6.5	164.2 ± 3.4	192.6 ± 11.2	0.5 ± 0.06
	**EAC**	165.8 ± 7.1^▀^^▀^^▀^	91.47 ± 4.9^▀^^▀^^▀^	127.8 ± 15.4^▀^^▀^	0.2 ± 0.02^▀^^▀^
	**DOX**	212 ± 7.6^▲▲▲^	88.39 ± 2	124.8 ± 9.3	0.1 ± 0.03
	**COE200**	105.8 ± 4.1^▲▲▲^	149.5 ± 9^▲▲▲^	181.9 ± 3.4^▲^	0.4 ± 0.03^▲^
	**COE200+ DOX**	168.5 ± 3.4^●●●^	131.7 ± 3.3^▲▲▲●●●^	176.2 ± 7.6^▲●^	0.3 ± 0.05
**Kidney**	**Normal**	252.9 ± 6.7	29.7 ± 2.4	46 ± 3.7	0.79 ± 0.02
	**EAC**	526.9 ± 18.9 ^▀^^▀^^▀^	13.03 ± 1.6 ^▀^^▀^^▀^	22.5 ± 2^▀^^▀^^▀^	0.38 ± 0.01^▀^^▀^^▀^
	**DOX**	483.1 ± 15.2 ^▲▲▲^	12.2 ± 0.4	14.9 ± 0.3	0.16 ± 0.01^▲▲▲^
	**COE200**	238 ± 13.2^▲▲▲^	26.2 ± 1.7 ^▲▲▲^	36.81 ± 2.8^▲▲^	0.60 ± 0.03^▲▲▲^
	**COE200+ DOX**	303.3 ± 11.9 ^▲▲▲^	20.41 ± 0.8^▲▲▲●●●^	21.7 ± 0.9	0.42 ± 0.0^●●●^

All values are expressed as a mean ± SEM (n = 6). One-way analysis of variance (ANOVA) followed by Tukey’s test.

^▀^^▀^^▀^ *p* < 0.001, compared with normal.

^▲^ *p* < 0.05.

^▲▲^ *p* < 0.01.

^▲▲▲^ *p* < 0.001, compared with EAC.

^●^ *p* < 0.05.

^●●^ *p* < 0.01.

^●●●^ *p* < 0.001, compared with DOX. LPO, lipid peroxidation; GSH, glutathione; SOD, superoxide dismutase; CAT: catalase.

#### Effect of COE on cardiac, hepatic, kidney, and solid tumor histology

**FIGURE 8:**
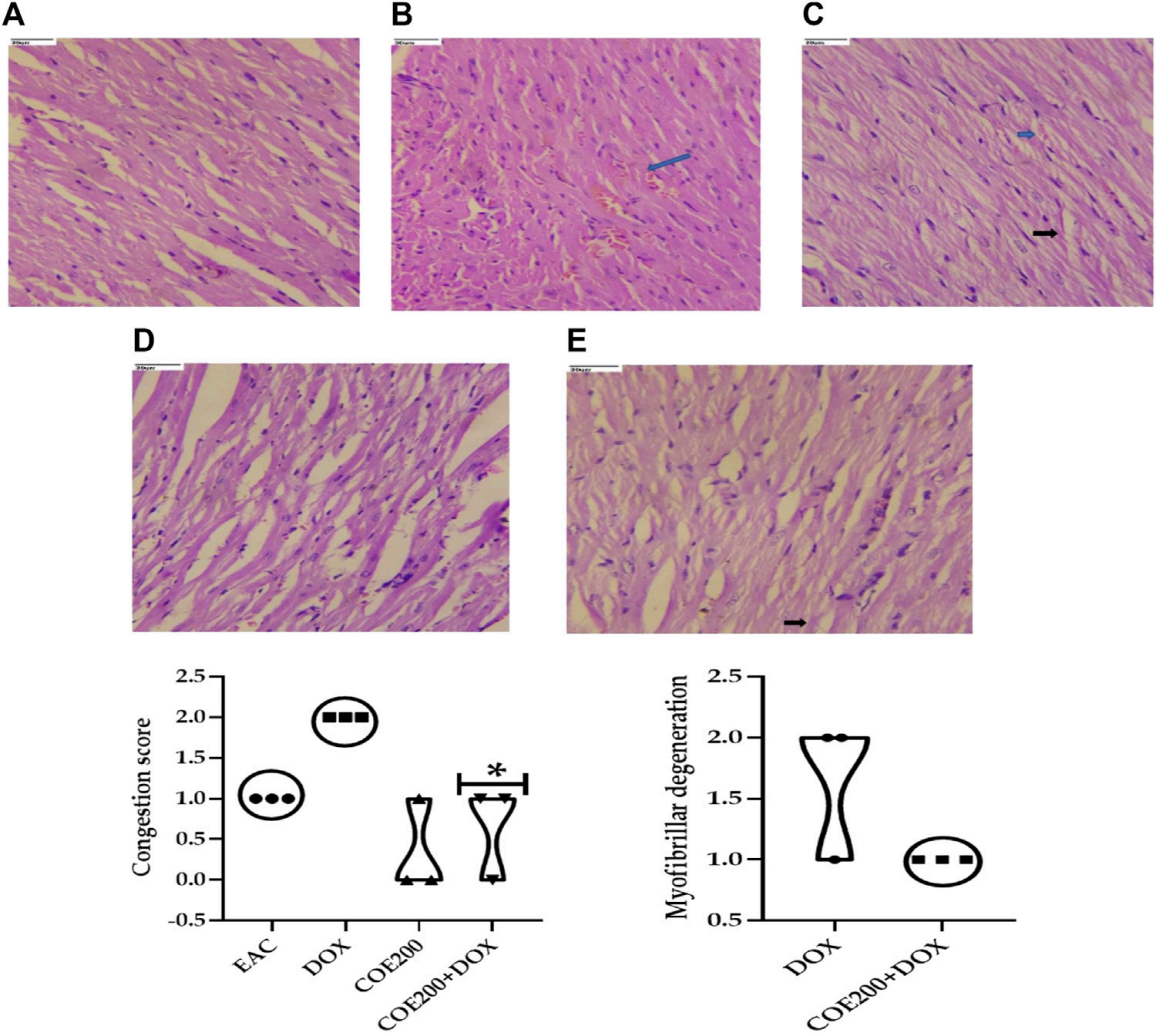
Effect of COE in cardiac histology. Photograph of the heart section of different treatment groups stained with hematoxylin and eosin. Plates at ×40 magnification. **(A)** Normal, **(B)** EAC, **(C)** DOX, **(D)** COE, and **(E)** COE + DOX. EAC group **(B)** showing congestion (blue arrow) and myofibrillar degeneration (black arrow). All values are expressed as mean ± SEM (n = 3), one-way analysis of variance (ANOVA) followed by Tukey’s test • *p* < 0.05, compared with DOX.

**FIGURE 9:**
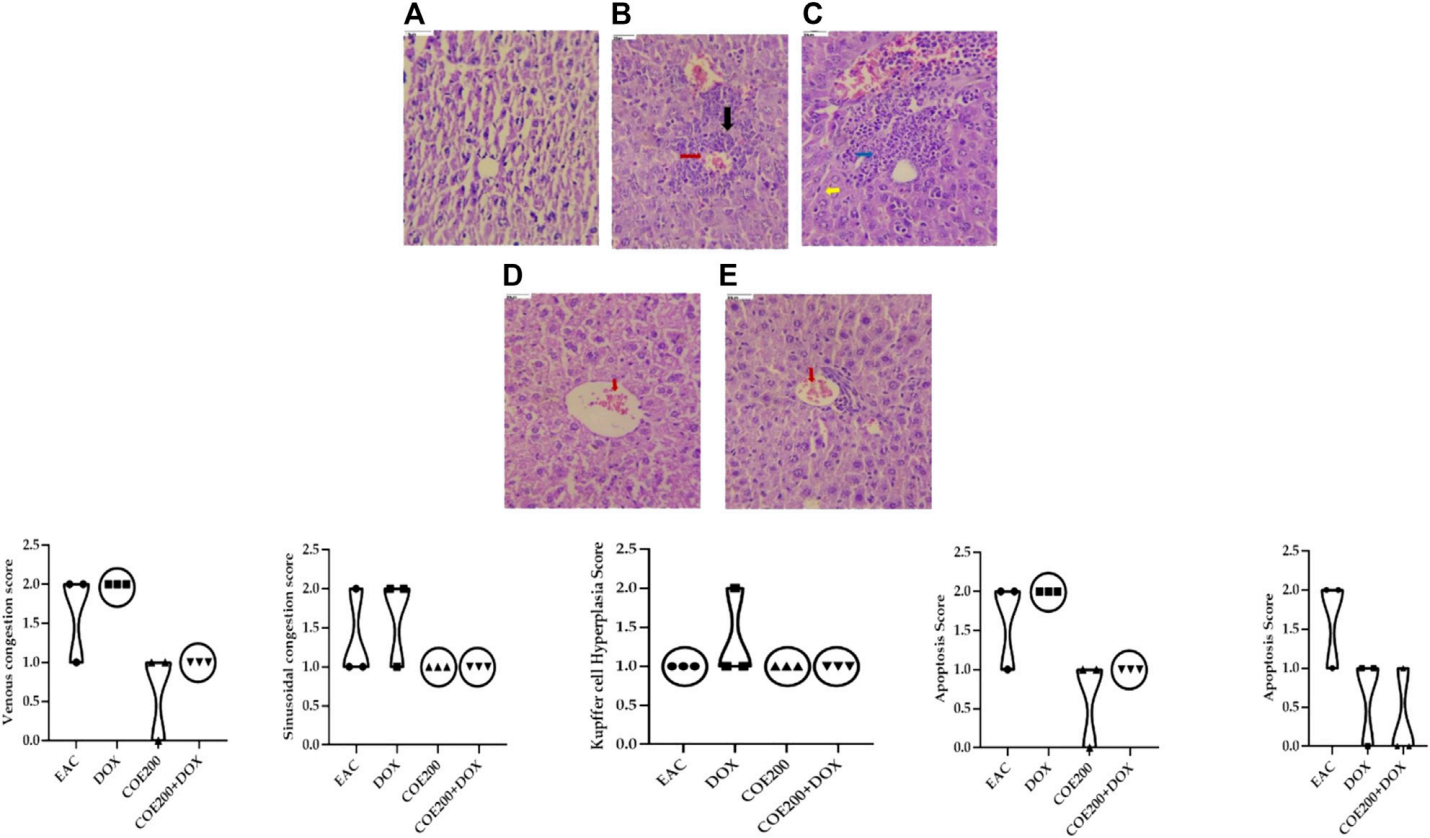
Effect of COE in hepatic histology. Photograph of liver section of different treatment groups stained with hematoxylin and eosin. Plates at ×40 magnification. **(A)** Normal, **(B)** EAC, **(C)** DOX, **(D)** COE, and **(E)** COE + DOX. EAC and DOX groups are showing venous and sinusoidal congestion (red and blue), Kupffer cell hyperplasia (yellow), apoptosis, and spotty necrosis (black). All values are expressed as a mean ± SEM (n = 3), one-way analysis of variance (ANOVA) followed by Tukey’s test.

**FIGURE 10:**
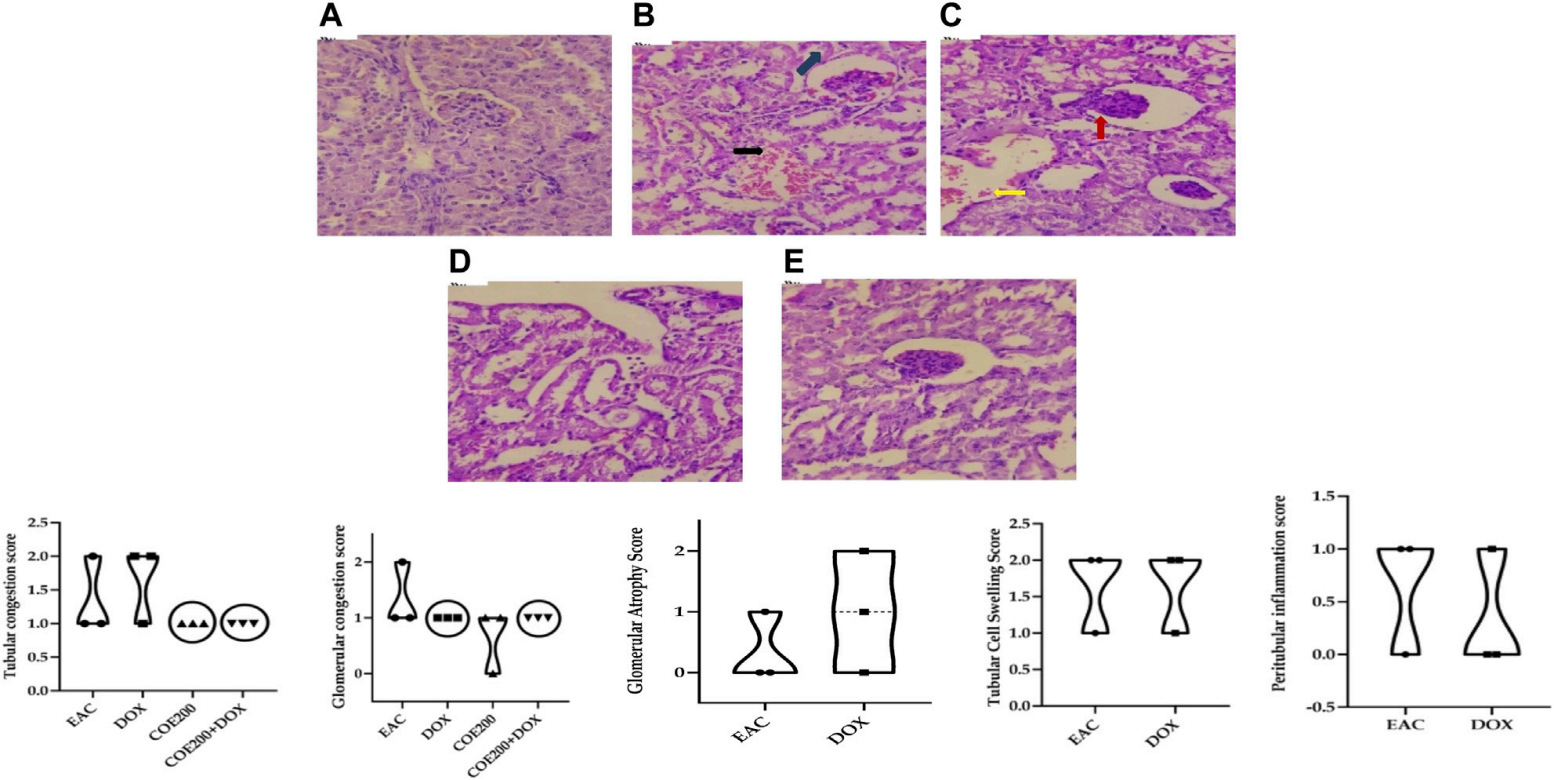
Effect of COE in kidney histology. Photograph of the kidney section of different treatment groups stained with hematoxylin and eosin. Plates at ×40 magnification. **(A)** Normal, **(B)** EAC, **(C)** DOX, **(D)** COE, **(E)** COE + DOX. EAC and DOX groups are showing tubular (black) and glomerular (blue) congestion, inflammation, glomerular atrophy (red), and tubular cell swelling (yellow). All values are expressed as a mean ± SEM (n = 3), one-way analysis of variance (ANOVA) followed by Tukey’s test.

**FIGURE 11:**
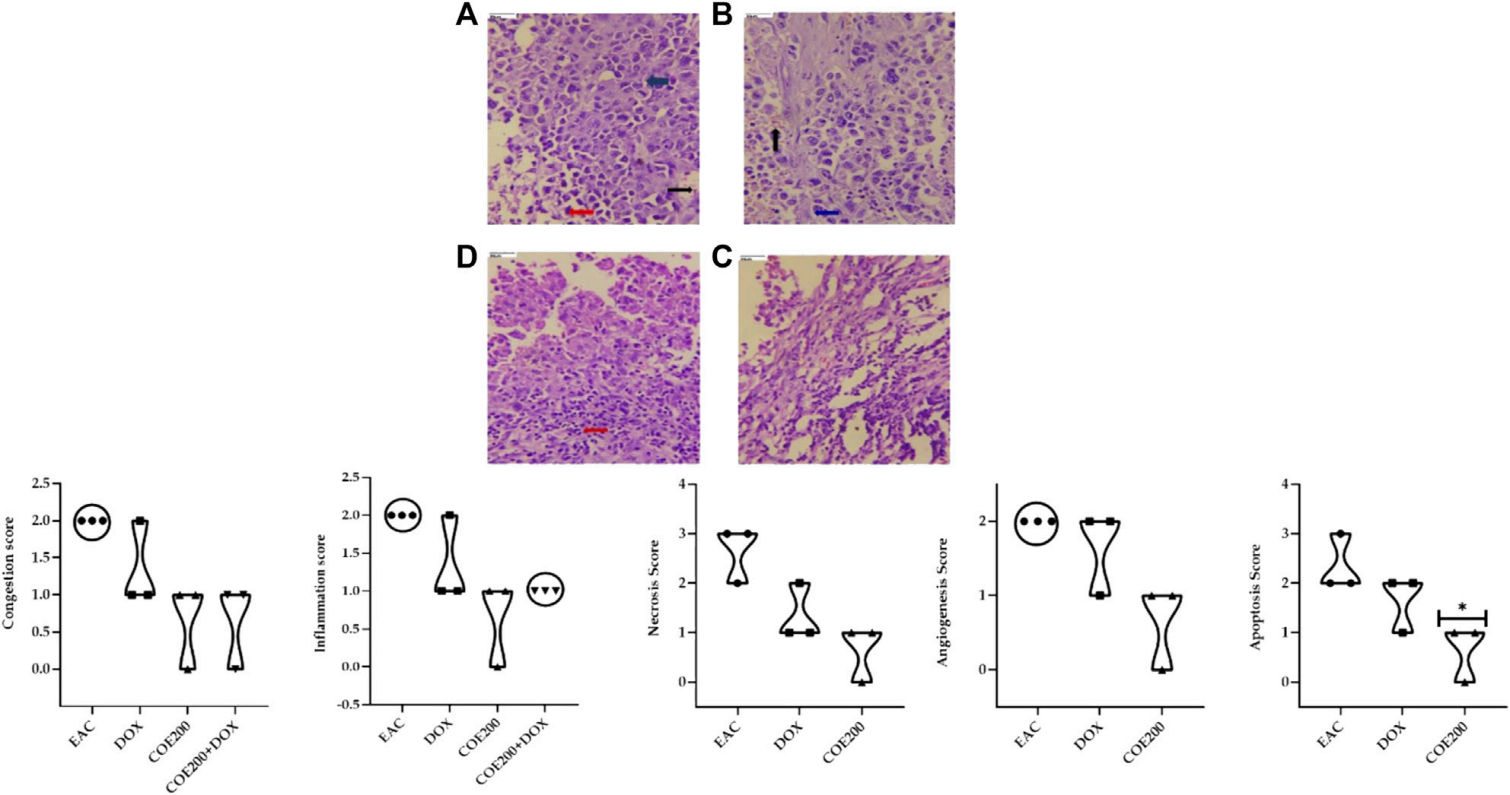
Effect of COE on tumor histology. Photograph of the tumor section of different treatment groups stained with hematoxylin and eosin. Plates at ×40 magnification. **(A)** EAC, **(B)** DOX, **(C)** COE, and **(D)** COE + DOX. EAC and DOX groups are showing congestion (black), inflammation (blue), hemorrhage, angiogenesis, apoptosis, and necrosis (red). All values are expressed as a mean ± SEM (n = 3). One-way analysis of variance (ANOVA) followed by Tukey’s test ^▲^ *p* < 0.05 compared with EAC.

Normal architecture was observed in the heart, liver, and kidney tissue in the normal group of animals. Furthermore, in the EAC group, congestion and myofibrillar degeneration were noted in cardiac tissues (Figure 8). Venous and sinusoidal congestion, Kupffer cell hyperplasia, spotty necrosis, apoptosis, inflammation, and hepatocellular dysplasia were noted within hepatic tissues (Figure 9), while tubular and glomerular congestion, glomerular atrophy, tubular cell swelling, inflammation, widening of the Bowman space, and cytoplasmic vacuoles were observed in kidney tissues (Figure 10). In addition, these variables were traced in the EAC group, which were markedly increased with doxorubicin-independent treatment. Furthermore, these histopathological changes were mitigated in COE-treated groups. We also observed the tumor tissue and saw a maximum number of cords and nests of tumor cells, congestion, angiogenesis, hemorrhage, invading muscle infiltration, fibrosis, necrosis, anaplasia, and mitotic activity in the EAC group compared to the rest of the interventions (Figure 11).

### *In silico* study

#### Molecular modeling studies

**FIGURE 12:**
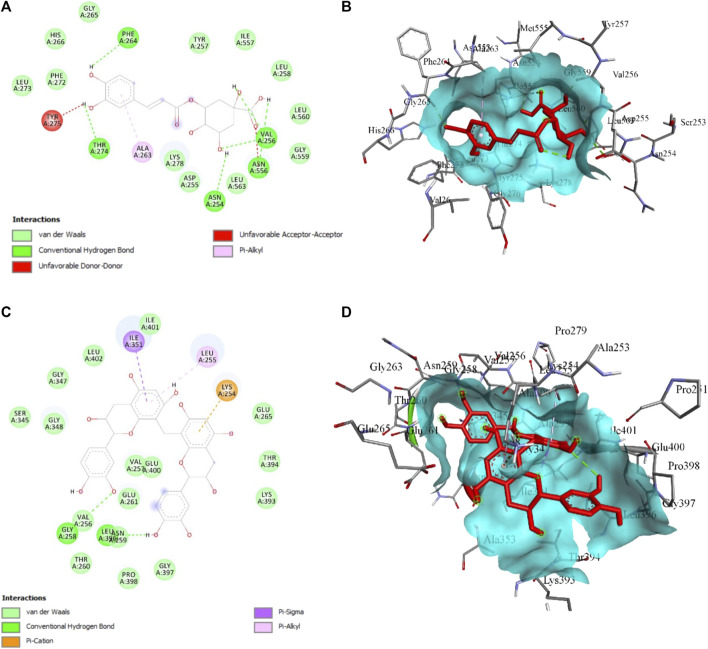
**(A)** 2D and **(B)** 3D presentation for the interaction of CHL with LOX, **(C)** 2D and **(B)** 3D presentation for the interaction of MBC with XO. The green bond represents the H-bond interaction, the red bond represents unfavorable interaction due to steric hindrance, and the rest represent hydrophobic interaction.

About 27 previously reported phytocompounds in cacao were collated (Patil et al., 2022b). The successful docking of these 27 phytocompounds to both the receptors LOX and XO has been performed using the POAP pipeline to the binding site determined by CastP. It has been observed that the 27 compounds docked to the LOX and XO bind to the binding pocket with varying binding affinity. We also observed that some of the phytocompounds bound to the LOX do not favor the binding site interactions and show higher binding energy (+ve binding energy value). The binding energy value of the phytocompounds bound to LOX ranged from −6.4 kcal/mol to + 38.5 kcal/mol, while phytocompounds bound to XO ranged from −6.2 kcal/mol to −11.3 kcal/mol. Careful analysis of the docked complexes revealed that large-sized compounds having a higher molecular weight failed to occupy the specified binding pocket in the LOX structure, and hence do not form stable interactions. We have now considered top-screened compounds, namely, chlorogenic acid (binding energy = −6.4 kcal/mol) and 8′8 methylenebiscatechin (binding energy = −11.3 kcal/mol) bound to LOX and XO, respectively, for further MD simulation studies. For simplicity, chlorogenic acid and 8′8 methylenebiscatechin are hereafter referred to as CHL and MBC, respectively. The orientation of the docked pose of complexes LOX–CHL and XO–MBC is shown in Figures 12A, B. The significant intermolecular non-bonded interactions stabilizing the complexes LOX–CHL and XO–MBC are shown in Table 4.

**TABLE 4:** Crucial intermolecular non-bonded interactions stabilizing LOX–CHL and XO–MBC complexes during MD simulation.

Complex	Conformation	Interactions	Type	Distance	Angle
LOX–CHL	Initial	B:UNL850:HO1–A:ASN556:OD1	Conventional hydrogen bond	2.91137	112.244
		B:UNL850:HO3–A:ARG252:O	Conventional hydrogen bond	2.41292	121.391
		A:LYS278:HE3–B:UNL850:O1	Carbon hydrogen bond	2.86753	129.811
		A:LEU560:HA–B:UNL850:O5	Carbon hydrogen bond	3.03038	110.953
		A:ASN254:HA–B:UNL850	Pi-sigma	2.74586	
		A:SER253:C,O; ASN254:N - B:UNL850	Amide-Pi stacked	4.39367	
	Final	B:UNL850:HO1–A:GLU197:OE1	Conventional hydrogen bond	1.47963	178.887
		B:UNL850:HO4–A:ASN556:O	Conventional hydrogen bond	2.92387	109.221
		A:GLY559:HA3–B:UNL850:O3	Carbon hydrogen bond	2.67742	127.64
		B:UNL850–A:LEU258	Pi-alkyl	5.41188	
XO–MBC	Initial	A:LYS247:HZ2–B:UNL1330:O4	Conventional hydrogen bond	1.91825	159.485
		A:LEU255:HN–B:UNL1330:O10	Conventional hydrogen bond	2.78719	153.983
		A:ILE351:HN–B:UNL1330:O9	Conventional hydrogen bond	3.02736	157.068
		A:SER353:HN–B:UNL1330:O7	Conventional hydrogen bond	2.31487	153.78
		A:ASN359:HD21–B:UNL1330:O7	Conventional hydrogen bond	2.00375	139.321
		B:UNL1330:HO3–A:GLY396:O	Conventional hydrogen bond	1.96909	133.105
		B:UNL1330:HO5–A:GLY347:O	Conventional hydrogen bond	1.71796	151.971
		B:UNL1330:HO6–A:ASN359:OD1	Conventional hydrogen bond	1.93259	138.227
		A:GLU252:HA–B:UNL1330:O11	Carbon hydrogen bond	2.89095	
		A:LYS254:HA–B:UNL1330:O10	Carbon hydrogen bond	2.46619	
		A:PRO397:HA–B:UNL1330:O3	Carbon hydrogen bond	2.6411	
		B:UNL1330:HC1–A:PRO251:O	Carbon hydrogen bond	2.71201	
		B:UNL1330:HO1–B:UNL1330	Pi-donor hydrogen bond	2.77657	
		B:UNL1330–A:ILE351	Alkyl	5.08421	
		B:UNL1330:C29–A:ILE351	Alkyl	4.4021	
		B:UNL1330–A:LEU395	Pi-alkyl		
	final	A:ILE350:HN–B:UNL1330:O9	Conventional hydrogen bond	1.91771	148.298
		A:ILE351:HN–B:UNL1330:O9	Conventional hydrogen bond	2.70033	170.438
		A:SER353:HN–B:UNL1330:O7	Conventional hydrogen bond	2.73856	157.904
		B:UNL1330:HO1–B:UNL1330:O	Conventional hydrogen bond	1.91953	164.544
		B:UNL1330:HO3–A:LEU395:O	Conventional hydrogen bond	1.93889	96.437
		B:UNL1330:HO4–A:LEU255:O	Conventional hydrogen bond	1.63738	168.378
		B:UNL1330:HO5–A:GLY347:O	Conventional hydrogen bond	1.88205	131.813
		B:UNL1330:HO8–A:ASN349:OD1	Conventional hydrogen bond	1.82011	
		B:UNL1330:HO8–A:ASN349:O	Conventional hydrogen bond	2.96447	
		B:UNL1330:HO9–A:GLU252:O	Conventional hydrogen bond	1.59684	
		A:PRO397:HA–B:UNL1330:O4	Carbon hydrogen bond	2.78821	
		A:LYS254:NZ–B:UNL1330	Pi-cation	4.3856	
		A:PHE273–B:UNL1330	Pi–Pi T-shaped	4.92736	
		B:UNL1330–A:ILE351	Alkyl	5.26081	
		B:UNL1330–A:LEU395	Pi-Alkyl	5.46509	
		B:UNL1330–A:LYS254			

**FIGURE 13:**
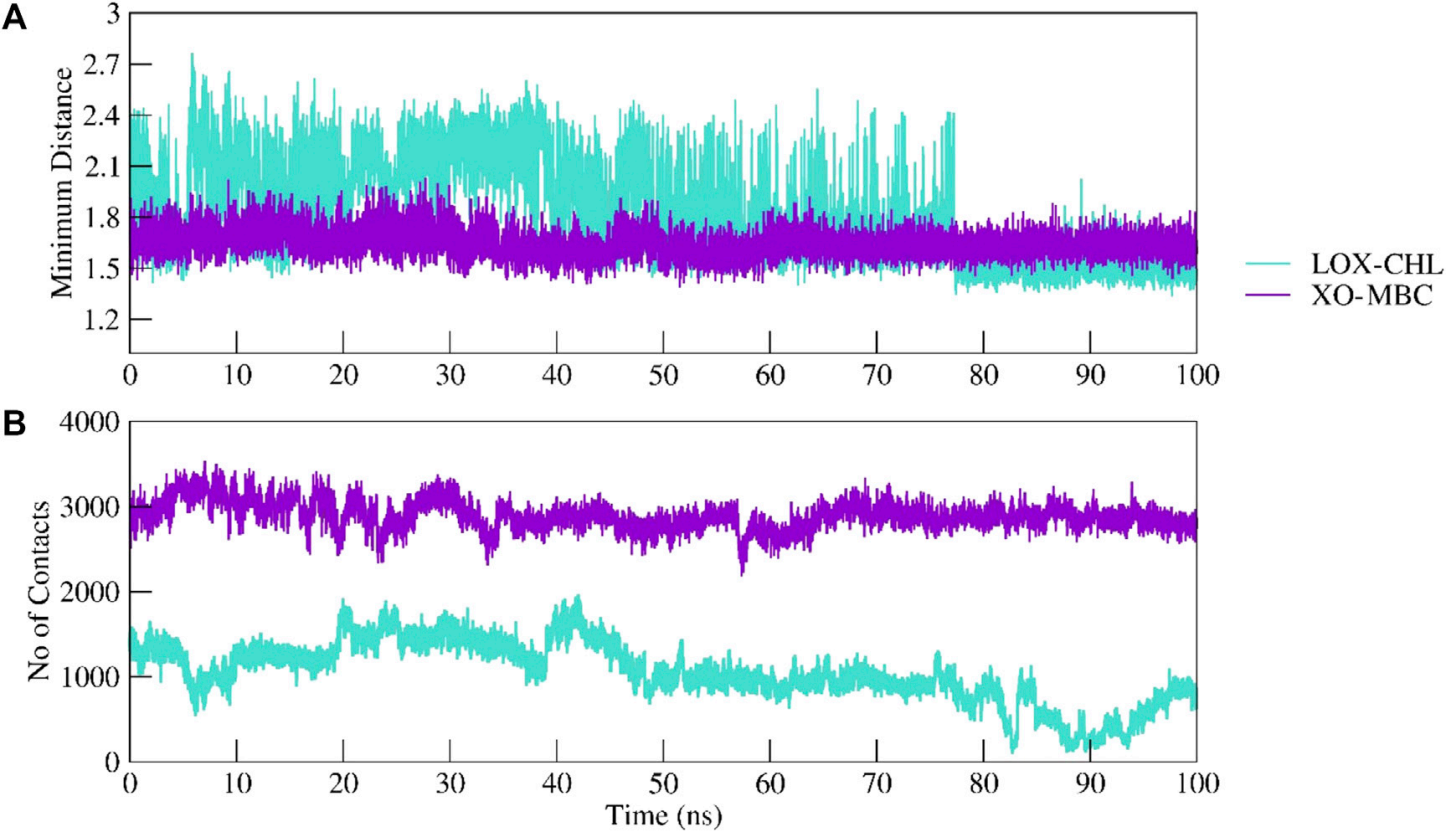
Intermolecular interactions between LOX–CHL and XO–MBC. Complexes were investigated by calculating the minimum distance between the protein and ligand group **(A)** and maximum number of total non-bonded contacts formed are shown **(B)**.

The structural stability of the complexes LOX–CHL and XO–MBC for the period of 100 ns has been investigated by analyzing parameters such as RMSD, RMSF, Rg, and SASA. The average RMSD values for complexes LOX–CHL and XO–MBC have been recorded as 1.8 Å and 3.2 Å, respectively (Supplementary Figure S1A). Both trajectories reached an equilibrated state after 40 ns of simulation. The LOX–CHL and XO–MBC complexes show very stable dynamics and conserved binding pocket interactions during the 100 ns of simulation (refer Supplementary Figures S1 and S2). The probability of RMSD distribution has been analyzed to gain more confidence in the convergence of the MD simulation trajectory (Supplementary Figure S2) The probability distribution plot showed a higher probability (0.9) for both the complexes LOX–CHL and XO–MBC for fewer RMSD values (1.8 Å and 3.25 Å, respectively), indicating the trajectories are well-converged (refer Supplementary Figure S2). The Rg values represent the compactness of both complexes and reveal the stable dynamics throughout the simulation (Supplementary Figure S1B). Similar stable dynamics have also been expressed by the SASA values representing the compact folding and showed no exposure of the binding pocket region to the solvent (Supplementary Figure S1B). The maximum number of H-bonding interactions formed by the complexes LOX–CHL and XO–MBC were 6 and 11, respectively (Supplementary Figure S1D). The RMSF values show the least residual fluctuations (<1.5 Å) at the active site residues and the surrounding region of the binding pocket in both complexes (Supplementary Figures S3A and S3B). In XO, the C-terminal flexible loop, where native secondary structure folds are lacking, represents maximum RMSF values (>6 Å). In LOX, the N-terminal loop region showed higher residual fluctuations up to 5 Å (Supplementary Figure S3A). The minimum distance and the total number of all contacts are plotted for both complexes to provide detailed insights into their inter-molecular interactions (Figures 13A, B).

The minimum distance plotted for LOX–CHL shows an increase in the distance of 2.4 Å; furthermore, it decreases to 1.5 after 80 ns, while XO–MBC shows a consistent minimum distance of 1.6 Å throughout the simulation period.

**FIGURE 14:**
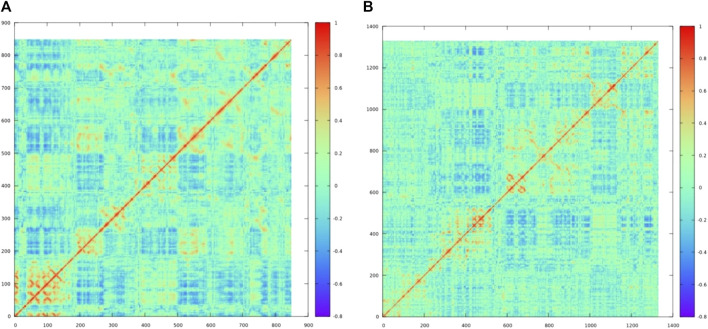
The dynamic cross-correlation matrix reveals concerted motion in the complexes **(A)** LOX–CHL and **(B)** XO–MBC. The red color represents the positive correlation, the blue color represents the negative correction, and the cyan color (zero value) represents no correlation.

The free energy landscape has been explored for both the complexes, of which complex XO–MBC showed the least energy state conformations were clustered in one group in the free energy landscape, while the other complex LOX–CHL showed three minimums over the free energy landscape, representing conformational diversity exhibited by the LOX upon binding of CHL. Supplementary Figures S4A and S4B represent the free energy landscape for both the complexes in 2D and 3D, respectively. Further concerted motion in complexes LOX–CHL (Figure 14A) and XO–MBC (Figure 14B) has been analyzed for representative frames from the stable trajectories, and it reveals the strong self-correlation for the individual residues (diagonal line shown in red) with itself, and binding pocket residues also express the positive correlation with moderate amplitude. Overall, the amplitude of the negative correlation was decreased in the complex XO–MBC when compared to LOX–CHL (Figures 14A, B).

## Discussion

The results obtained during our study clearly demonstrated that COE alone or in combination with doxorubicin exerted better antitumor activity than doxorubicin alone, as evidenced by the improved survival time and tumor regression. *In vitro* assays provide the physiological environment to examine several cellular activities. *In vitro* studies showed that COE alone has a role in the reduction of the cell proliferation rate, colony formation capability, and migratory potential in EAC as well as human cancer cell lines (A549). COE has demonstrated stronger anticancer action on cancer cells than on healthy cells. In addition, COE enhanced the potency of doxorubicin when used in combination with a lower concentration (of doxorubicin) in the inhibition of cancer cell proliferation, migration, and cell cycle arrest. In this assay, the 1:1 ratio of DOX and cocoa (50% of each) in the combination group was selected to evaluate the potential therapeutic benefits of DOX at a lower concentration when used in combination with cocoa. Furthermore, it also has the added advantage of preventing possible undesirable effects when combining the two substances at higher concentration. The scratch assay results suggest that COE might have a role in the inhibition of epithelial–mesenchymal transition (EMT), the first step of metastasis. The induction of the EMT process leads to resistance toward chemotherapeutic drugs (Shibue and Weinberg, 2017). The EMT induction may reduce the cell proliferation rate and increase the expression of proteins responsible for inhibiting apoptosis as well as the expression of transporter-related proteins to efflux drugs (Shibue and Weinberg, 2017). The COE might be involved in the modulation of EMT initiation. Some anti-proliferative agents have already been designed to inhibit EMT initiation (Thiery and Sleeman, 2006). The natural product COE can be used as an alternative anti-proliferative agent. The COE may be involved in the modulation of signaling processes and may provide a better delivery system for chemotherapeutic drugs such as doxorubicin. However, further experimental validation will be needed to confirm our hypothesis.

Furthermore, COE markedly reduced the doxorubicin-induced cytotoxicities in cardiac, hepatic, and nephritic tissues in the EAC-induced solid tumor mouse model. The cell protective nature of COE toward cardiac myocytes, hepatocytes, and nephrons, manifested through histological and biochemical investigation, could be through the termination of Fenton and redox reactions, as there were ameliorated enzymatic and non-enzymatic antioxidant biomarkers.

Further combinatorial therapy of COE with doxorubicin in tumor-bearing mice showed an increase in survival time by 1.20 fold compared to the DOX group. Within 21 days of treatment, we observed a significant decrease in tumor size in doxorubicin- and COE-treated animals; the outcome was further effective with combined treatment, which was evidenced via the assessment of tumor size. This decrease in tumor size with combined treatment could be the outcome of the synergetic effect, which might be due to the anti-cancer potency of COE (Martin et al., 2013). Moreover, there was a significant increase in the percentage change in body weight in the EAC group, which pointedly declined with the combined and independent action of both agents. In addition, there was no significant difference between the percentage change in body weight in the doxorubicin- and COE-treated groups. However, the percentage change in body weight was significantly lower in combination than in the DOX group, suggesting COE’s synergetic effect with doxorubicin. In addition, this decrease in percentage change in body weight supports the outcome of controlling tumor size with each intervention. Likewise, we observed a significant reduction in tumor weight with doxorubicin and COE-independent and combination action over tumor weight; a significant reduction in tumor weight in combination vs*.* DOX points toward a synergistic effect. Furthermore, the anti-tumor effect of COE reflected in our study is the auxiliary outcome, conveyed via a significant increase in lifespan.

In chemotherapy, doxorubicin has a major role in dealing with many cancers. However, it produces severe toxic effects on hematological parameters, which is one of the limiting factors in implementing doxorubicin chemotherapy (Ogura, 2001). In this regard, one can contemplate if any natural products with antioxidant potency could ameliorate the hematological parameters over synthetic chemotherapeutic agents, including doxorubicin. Herein, we observed significant amelioration of multiple hematological parameters with the combined action of COE with doxorubicin vs*.* DOX. Previously, it was reported that doxorubicin metabolites disturb hematological function, which could be a doxorubicin-activated ROS system (Afsar et al., 2017). In addition, ROS has a direct impact on Hb, RBC, WBC, platelets, and other hematological parameters, which directly affects cellular apoptosis due to compromising immunity, affecting the transport of nutrition and oxygen (Gwozdzinski et al., 2021). Previously, COE extract was reported for its potent antioxidant activity due to the presence of phenolic bioactives and flavonoids (catechin, epicatechin, and procyanidins) (Lamuela-Raventós et al., 2001; Massee et al., 2015); this may trigger scavenging free radicals and terminate redox and Fenton reactions within the ROS system, which could have driven the ameliorated hematological parameters.

In cancer chemotherapy, organ toxicity is one of the major complications, and it affects the homeostatic function, which is most commonly reported in doxorubicin therapy (Mitry and Edwards, 2016; Santos and Goldenberg, 2018). Therefore, we quantified multiple parameters like CK-MB, LDH, AST, ALT, ALP, creatinine, and BUN in different treatment groups. We found that these biomarkers were significantly increased in treatment with doxorubicin alone compared to EAC, indicating chemotherapy-induced organ toxicities. Likewise, free radical generation during doxorubicin therapy causes significant damage to the myocardium, resulting in increased membrane permeability and release of CPK-MB and LDH enzymes. Doxorubicin treatment showed a 1.43-fold and 1.23-fold increase in CPK_MB and LDH enzymes, respectively, compared to EAC treatment. In addition, the liver and kidney showed an increased AST level by 1.44 folds, ALT by 1.55 folds, creatinine by 1.14 folds, and BUN by 1.13 folds compared to that of EAC. Interestingly, these parameters were markedly reversed in a combinatorial regimen of COE along with doxorubicin, which showed better activity of COE in the protection of vital organs in chemotherapy. This protective action of cocoa is owing to its defense against oxidative stress induced during chemotherapy. Cocoa in the form of dark chocolate high in flavonoids may be a good strategy for reducing cardiovascular risk by having beneficial effects in inhibiting platelet aggregation, lowering blood pressure, reducing dyslipidemia, and lowering plasma glucose levels (Zięba et al., 2019). Reactive oxygen species, which are generated during drug biotransformation processes, can bind and react with cellular components in the liver to cause liver damage and thereby impair liver function (Cichoż-Lach and Michalak, 2014). Antioxidants present in cocoa increase nitric oxide levels and have hepatoprotective potential (Asiedu-Gyekye et al., 2016)**.** A similar trend was observed when antioxidant biomarkers were monitored. The elevated levels of LPO and decreased GSH, SOD, CAT, and total thiol levels in the DOX group compared to the EAC group were substantially reversed in all COE-treated groups, augmenting its effectiveness in organ protection. Similar findings were reported previously for the potency of COE in the neutralization of the ROS system generated via various stress responses that could have been ameliorated, as evidenced by multiple investigations into regulating the homeostatic functions of various organs (S. Noori et al., 2008).

Lipoxygenase and xanthine oxidase are involved in the generation of oxidative stress (Chung et al., 1997; Wisastra and Dekker, 2014), which is observed in various pathogenic conditions, including cancer, and contributes to tissue injury (Pizzino et al., 2017). In the present study, we observed the amelioration of various oxidative stress biomarkers in multiple organs after COE co-treatment with doxorubicin. Moreover, our previous studies also support the efficacy of COE in ameliorating oxidative stress and cancer progression (Patil et al., 2022b; 2022a). Emmanuel et al. reported that cocoa leaf polyphenolic-rich extract inhibited xanthine oxidase due to the flavonoids and phenolic acids present in the extract (Irondi et al., 2017). In addition, catechin and its derivatives are reported to inhibit xanthine oxidase (catechin 303.95 μM, uncompetitive; epicatechin 20.48 μM, mixed; epigallocatechin 10.66 μM, mixed; epicatechin gallate 2.86 μM, mixed; and epigallocatechin gallate 0.76 μM, competitive inhibition. Collectively, cocoa flavonoids not only have antioxidant effects but also inhibit lipoxygenase activities (Schewe et al., 2002). Human LOX5 and LTA4 synthase activities are inhibited by (−)-epicatechin, and its low-molecular-weight procyanidins from cocoa products have a purported anti-inflammatory effect (Schewe et al., 2002). Previous studies showed CHL as a potent LOX inhibitor with an IC_50_ of 0.32 mg/mL and 61.27 μmol/L. In addition, based on the molecular docking and MD simulations, we observed chlorogenic acid and 8′8 methylenebiscatechin to have the highest binding affinity with lipoxygenase and xanthine oxidase to support their potential in ameliorating oxidative stress, which is also supported by the previous reports of the anti-oxidant properties of chlorogenic acid (Xu et al., 2012) and catechins (Bernatoniene and Kopustinskiene, 2018).

The roles of the aforementioned markers were further supported by the results of histopathological examination. There was a significant increase in the congestion score and myofibrillar degeneration in the EAC solid tumor model, which was further increased with doxorubicin treatment. Similar findings of cardiotoxicity were reported by Reddy et al. (2012), in which the authors demonstrated the efficacy of the cocoa extract as a cardioprotective agent in the EAC solid tumor model. Furthermore, in the EAC and DOX groups, we traced a remarkable increase in spotty necrosis, apoptosis, inflammation, hepatocellular dysplasia, venous and sinusoidal congestion, and Kupffer cell hyperplasia in hepatic tissues (Reddy et al., 2012). However, in COE 200, regeneration of cells was noted; this compromised tissue damage. These results summarize the effect of COE on doxorubicin-induced organ toxicity. Similarly, EAC- and DOX-exposed groups pointed toward tubular and glomerular congestion, glomerular atrophy, tubular cell swelling, inflammation, widening Bowman space, and cytoplasmic vacuoles, which were ameliorated via COE treatment, whether independent or in combination with doxorubicin. Furthermore, the observed cytotoxicity was also confirmed by histopathological devastation of the tumor mass, reduced tumor growth, decreased mitotic pattern, increased necrosis, and the occurrence of apoptotic nuclei via COE treatment.

## Conclusion

This study not only demonstrated the protective effect of COE against doxorubicin-induced organ toxicities (heart, liver, and kidney) but also indicated synergistic potential with the anticancer activity of doxorubicin. Furthermore, our study demonstrated the efficacy of COE to neutralize the free radicals generated by doxorubicin; maintain cell integrity, along with inherent anti-cancer properties; and prolong the survival time of EAC mice. Overall, COE exhibits promising nutraceutical properties toward cardioprotective, hepatoprotective, and nephroprotective effects when supplemented with doxorubicin. Further confirmatory studies at the clinical level are needed to establish COE as a health supplement in cancer patients undergoing doxorubicin-based chemotherapy.

## Data Availability

The original contributions presented in the study are included in the article/Supplementary Materials; further inquiries can be directed to the corresponding authors.
