# Supplementary material for: Computational and experimental pharmacology to decode the efficacy of Theobroma cacao L. against doxorubicin-induced organ toxicity in EAC-mediated solid tumor-induced mice
Source: Front Pharmacol. 2023 May 31;14:1174867. doi: 10.3389/fphar.2023.1174867 (PMC10264642; doi:10.3389/fphar.2023.1174867)
Supplement: Supplementary file 1 [file Presentation1.PPTX]

## Slide 1
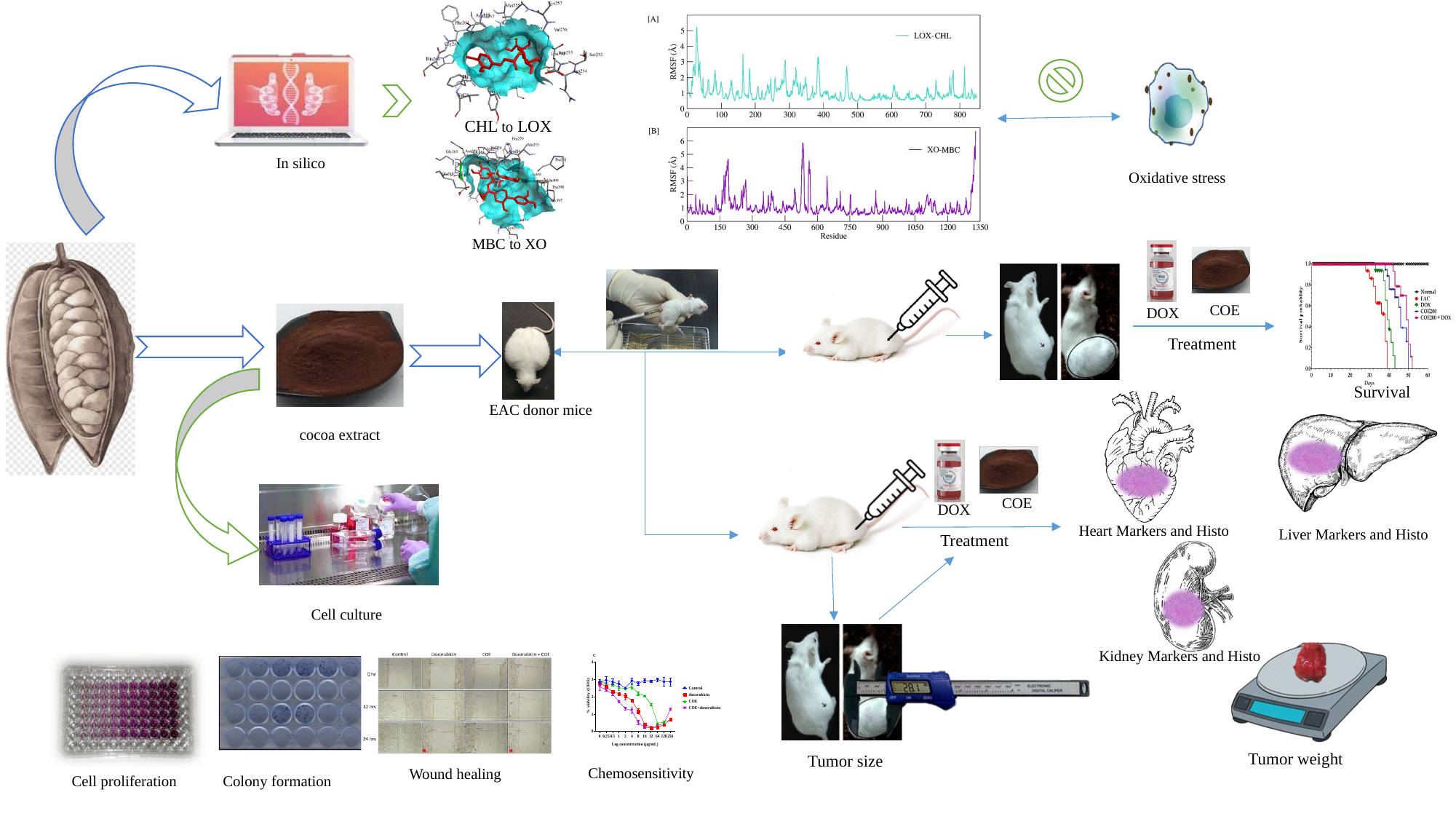

CHL to LOX
In silico
Oxidative stress
MBC to XO
COE
DOX
Treatment
Survival
EAC donor mice
cocoa extract
COE
DOX
Heart Markers and Histo
Liver Markers and Histo
Treatment
Cell culture
Kidney Markers and Histo
Tumor weight
Tumor size
Chemosensitivity
Wound healing
Cell proliferation
Colony formation
