# Supplementary material for: Computational and experimental pharmacology to decode the efficacy of Theobroma cacao L. against doxorubicin-induced organ toxicity in EAC-mediated solid tumor-induced mice
Source: Front Pharmacol. 2023 May 31;14:1174867. doi: 10.3389/fphar.2023.1174867 (PMC10264642; doi:10.3389/fphar.2023.1174867)
Supplement: Supplementary file 4 [file DataSheet1.docx]

Supplementary file


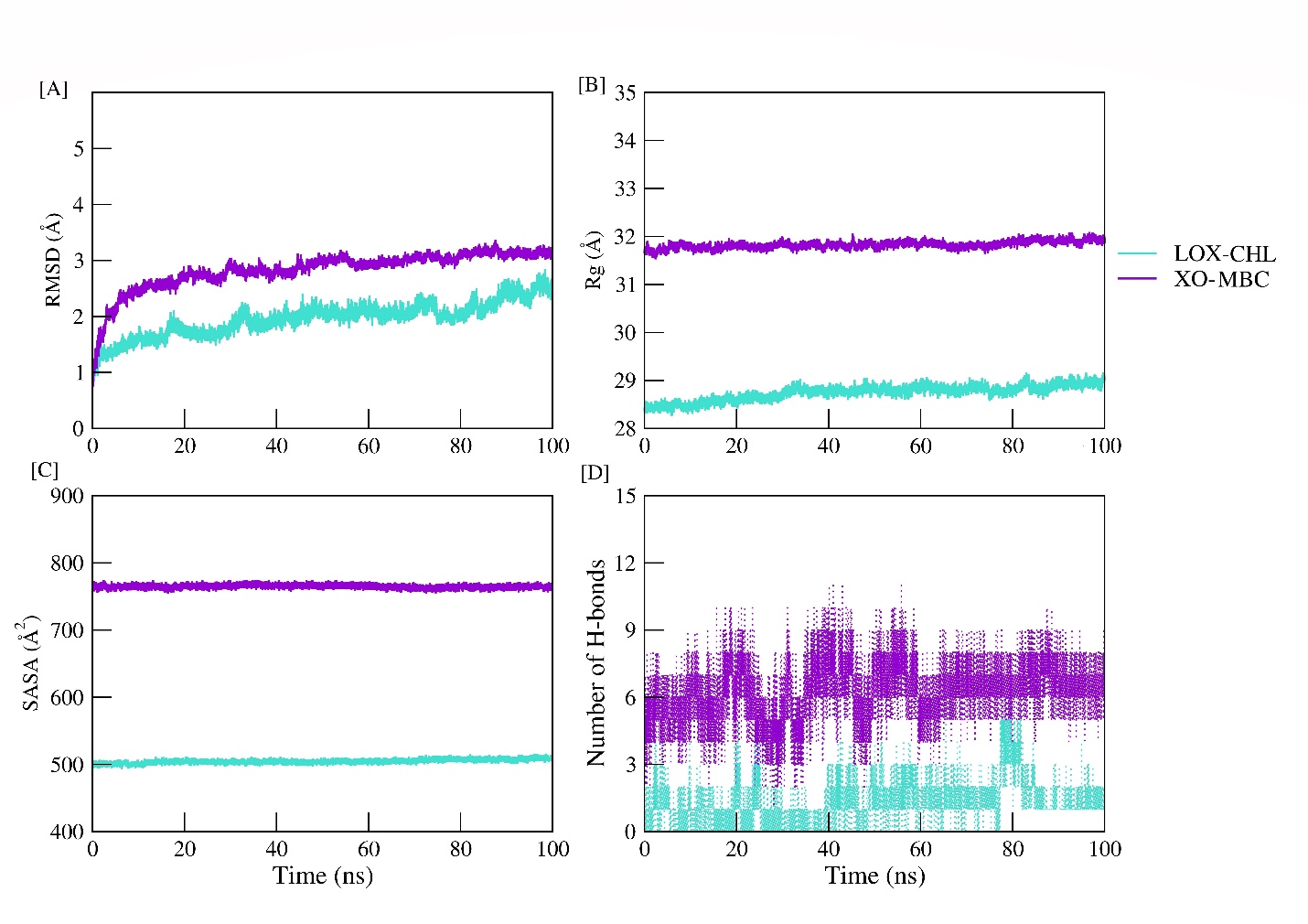


***Supplementary Figure S1:***The parameters explaining structural stability of LOX-CHL and XO-MBC complexes such as RMSD (A), Rg (B), SASA (C) and Number of H-bonds (D) are shown.


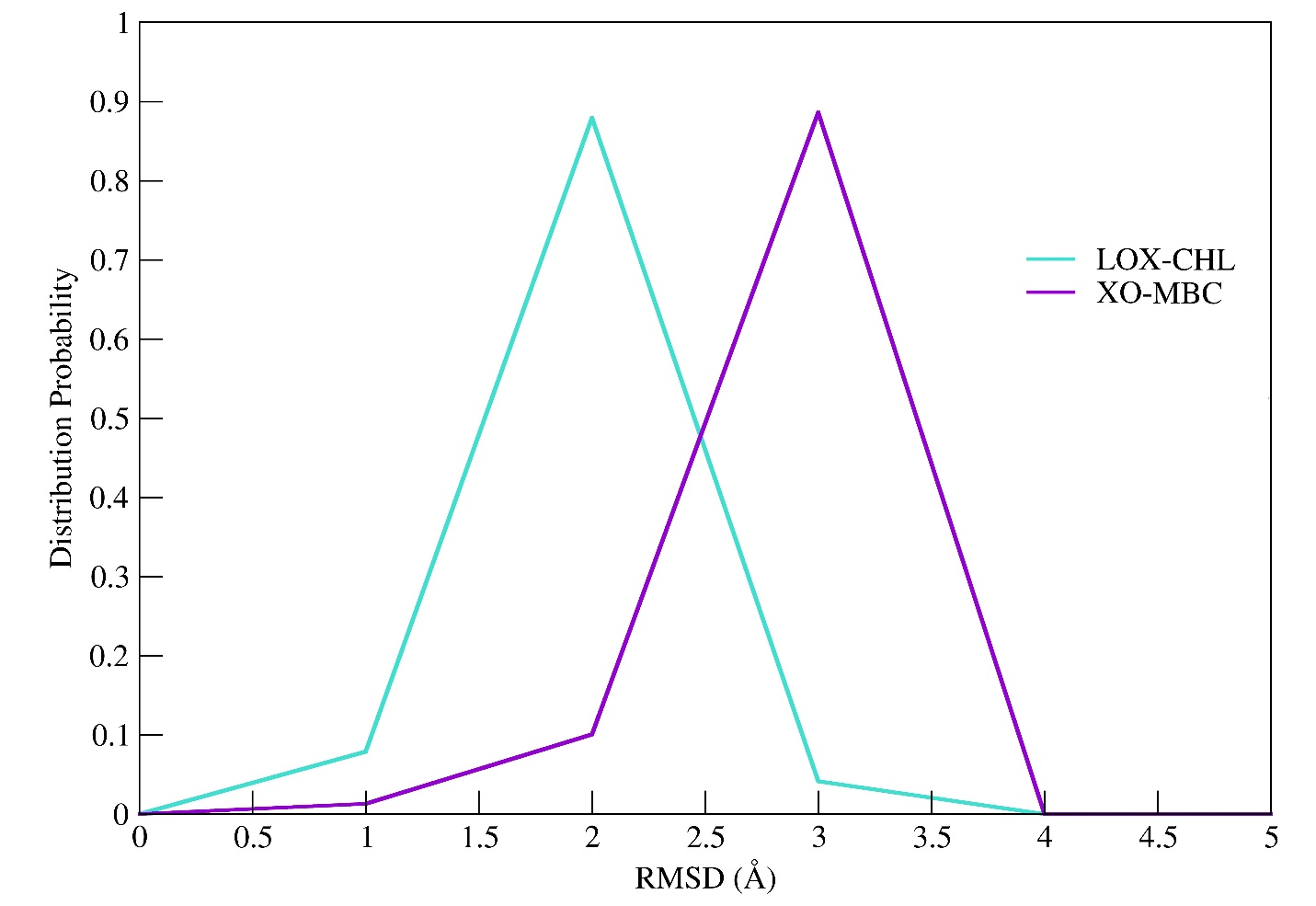


***Supplementary Figure S2:***The probability distribution of RMSD values for complexes LOX-CHL and XO-MBC is shown.


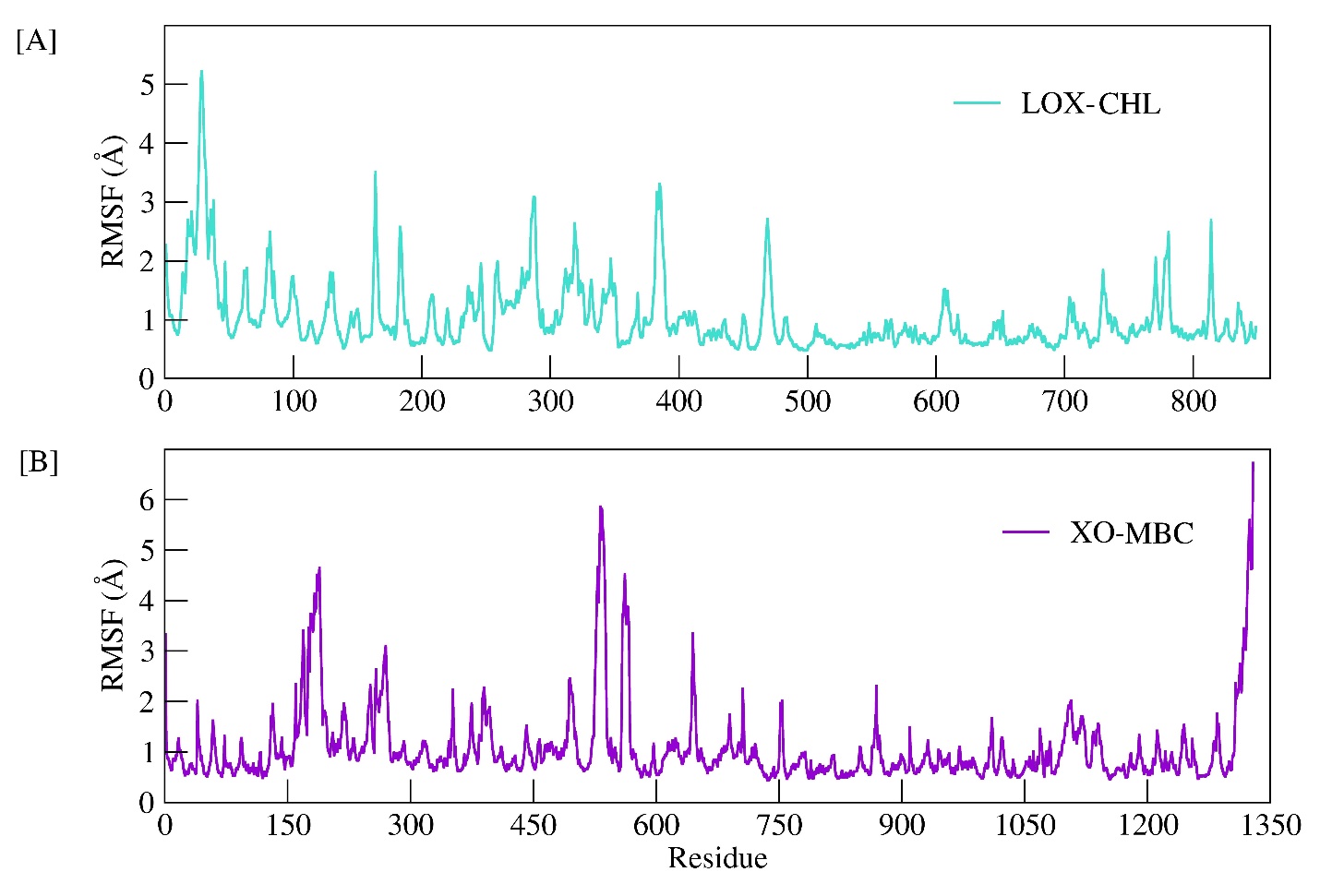


***Supplementary Figure S3:***The residual fluctuations in the complex LOX -MBC (A) and XO-CHL (B) during the MD simulation is shown.


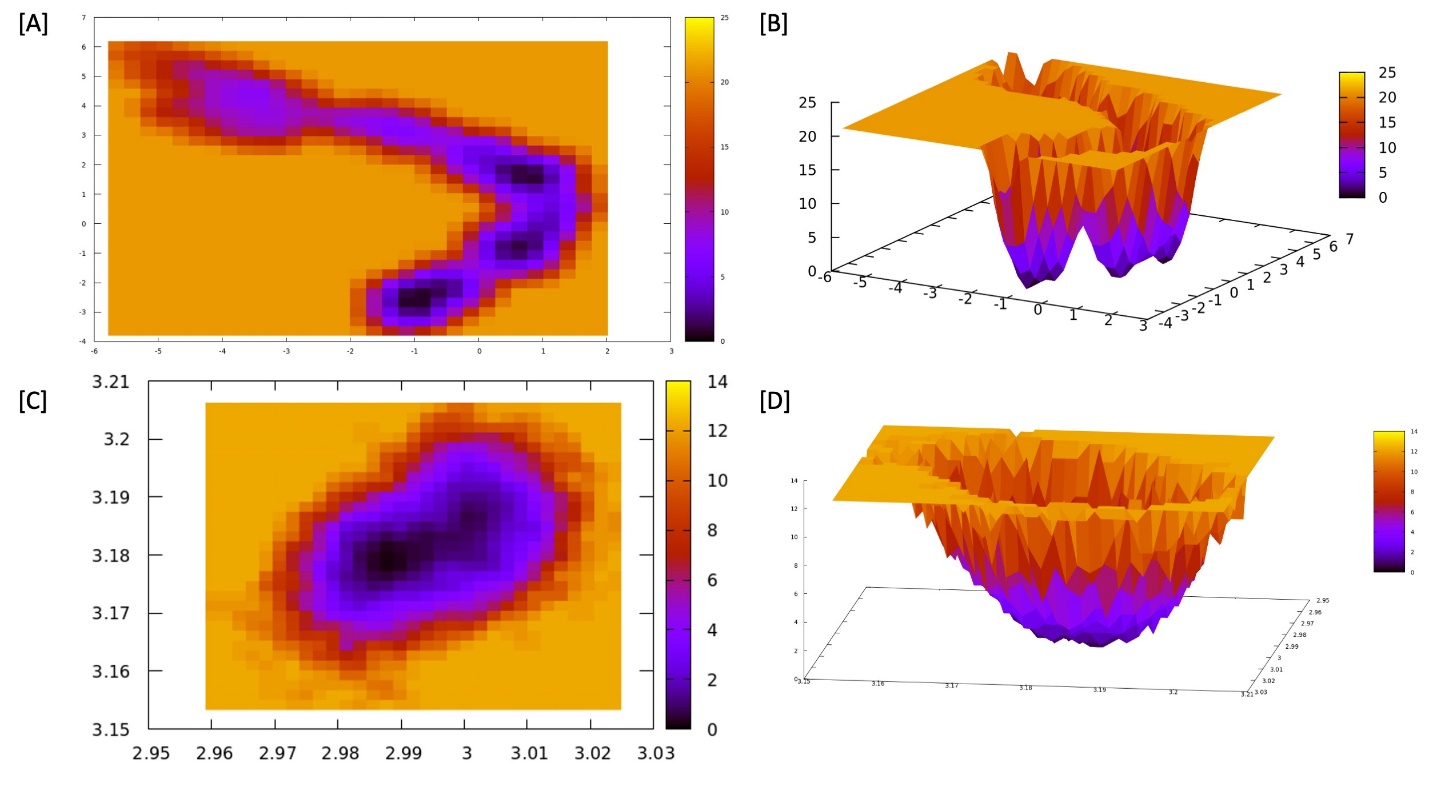


**Supplementary Figure S4:**The Free energy landscape generated in 2D as well as 3D to explore potential energy surfaces for complexes LOX-CHL (A and B) and XO-MBC (C and D).
